# Enhanced weathering in the US Corn Belt delivers carbon removal with agronomic benefits

**DOI:** 10.1073/pnas.2319436121

**Published:** 2024-02-22

**Authors:** David J. Beerling, Dimitar Z. Epihov, Ilsa B. Kantola, Michael D. Masters, Tom Reershemius, Noah J. Planavsky, Christopher T. Reinhard, Jacob S. Jordan, Sarah J. Thorne, James Weber, Maria Val Martin, Robert P. Freckleton, Sue E. Hartley, Rachael H. James, Christopher R. Pearce, Evan H. DeLucia, Steven A. Banwart

**Affiliations:** ^a^Leverhulme Centre for Climate Change Mitigation, School of Biosciences, University of Sheffield, Sheffield S10 2TN, United Kingdom; ^b^Institute for Sustainability, Energy, and Environment, University of Illinois at Urbana-Champaign, Urbana, IL 61801; ^c^Yale Center for Natural Carbon Capture, Department of Earth & Planetary Sciences, Yale University, New Haven, CT 06511; ^d^School of Earth and Atmospheric Sciences, Georgia Institute of Technology, Atlanta, GA 30332; ^e^Mati Carbon, Houston, TX 77019; ^f^School of Ocean and Earth Science, National Oceanography Centre Southampton, University of Southampton, Southampton SO14 3ZH, United Kingdom; ^g^National Oceanography Centre, Southampton SO14 3ZH, United Kingdom; ^h^Global Food and Environment Institute, University of Leeds, Leeds LS2 9JT, United Kingdom; ^i^School of Earth and Environment, University of Leeds, Leeds LS2 9JT, United Kingdom

**Keywords:** agricultural production, carbon removal, enhanced weathering, soil geochemistry

## Abstract

Terrestrial enhanced weathering (EW) of silicate rocks, such as crushed basalt, on farmlands is a promising scalable atmospheric carbon dioxide removal (CDR) strategy that urgently requires performance assessment with commercial farming practices. We report findings from a large-scale replicated EW field trial across a typical maize-soybean rotation on an experimental farm in the heart of the United Sates Corn Belt over 4 y (2016 to 2020). We show an average combined loss of major cations (Ca^2+^ and Mg^2+^) from crushed basalt applied each fall over 4 y (50 t ha^−1^ y^−1^) gave a conservative time-integrated cumulative CDR potential of 10.5 ± 3.8 t CO_2_ ha^−1^. Maize and soybean yields increased significantly (*P* < 0.05) by 12 to 16% with EW following improved soil fertility, decreased soil acidification, and upregulation of root nutrient transport genes. Yield enhancements with EW were achieved with significantly (*P* < 0.05) increased key micro- and macronutrient concentrations (including potassium, magnesium, manganese, phosphorus, and zinc), thus improving or maintaining crop nutritional status. We observed no significant increase in the content of trace metals in grains of maize or soybean or soil exchangeable pools relative to controls. Our findings suggest that widespread adoption of EW across farming sectors has the potential to contribute significantly to net-zero greenhouse gas emissions goals while simultaneously improving food and soil security.

## Significance

Safe, scalable atmospheric carbon dioxide removal (CDR) strategies are required for addressing the current climate emergency alongside dramatic greenhouse gas emissions reductions. Enhanced weathering (EW) is a CDR strategy that involves amending farmland soils with crushed silicate rock, typically basalt, a common volcanic rock. Our results from a long-term, large-scale EW field trial in the United States Corn Belt demonstrate reproducible carbon removal on farm fields, alongside increased soil fertility and crop yield. Our findings highlight the substantial untapped potential for utilizing millions of hectares of US farmland to scale EW and deliver CDR with sustainable food and biofuel production.

Large-scale deployment of atmospheric carbon dioxide removal (CDR) strategies alongside emissions reductions will be essential for limiting future climate change caused by anthropogenic emission of CO_2_ and other greenhouse gases (1). Terrestrial enhanced weathering (EW), the amendment of cropland soils with crushed silicate rocks, such as basalt, is a promising CDR strategy (2–5). Purposeful EW accelerates dissolution of rock minerals to release cations and convert atmospheric CO_2_ into bicarbonate ions (HCO_3_^−^) which are stored in groundwater and oceans on a >10,000-y timescale (2–4). Biogeochemical modeling suggests that deployment of EW with basalt across major agricultural regions worldwide could sequester up to two billion metric tons of CO_2_ annually, after accounting for operational carbon emissions (mining, grinding, transport, and spreading of rock dust on fields) (4). In contrast to many other CDR strategies, EW can improve food security and soil health (3, 6) and reduce ocean acidification (7–9). Importantly, EW utilizes existing technology and infrastructure making it a rapidly scalable CDR option for assisting with national net-zero greenhouse gas emission plans (10). However, we urgently need to measure rates of EW at the farm scale using basalt in key agricultural regions over multiple years, with assessment of yield responses of different crop types, and soil biogeochemistry of inorganic nutrients and trace metals.

**Fig. 1.**
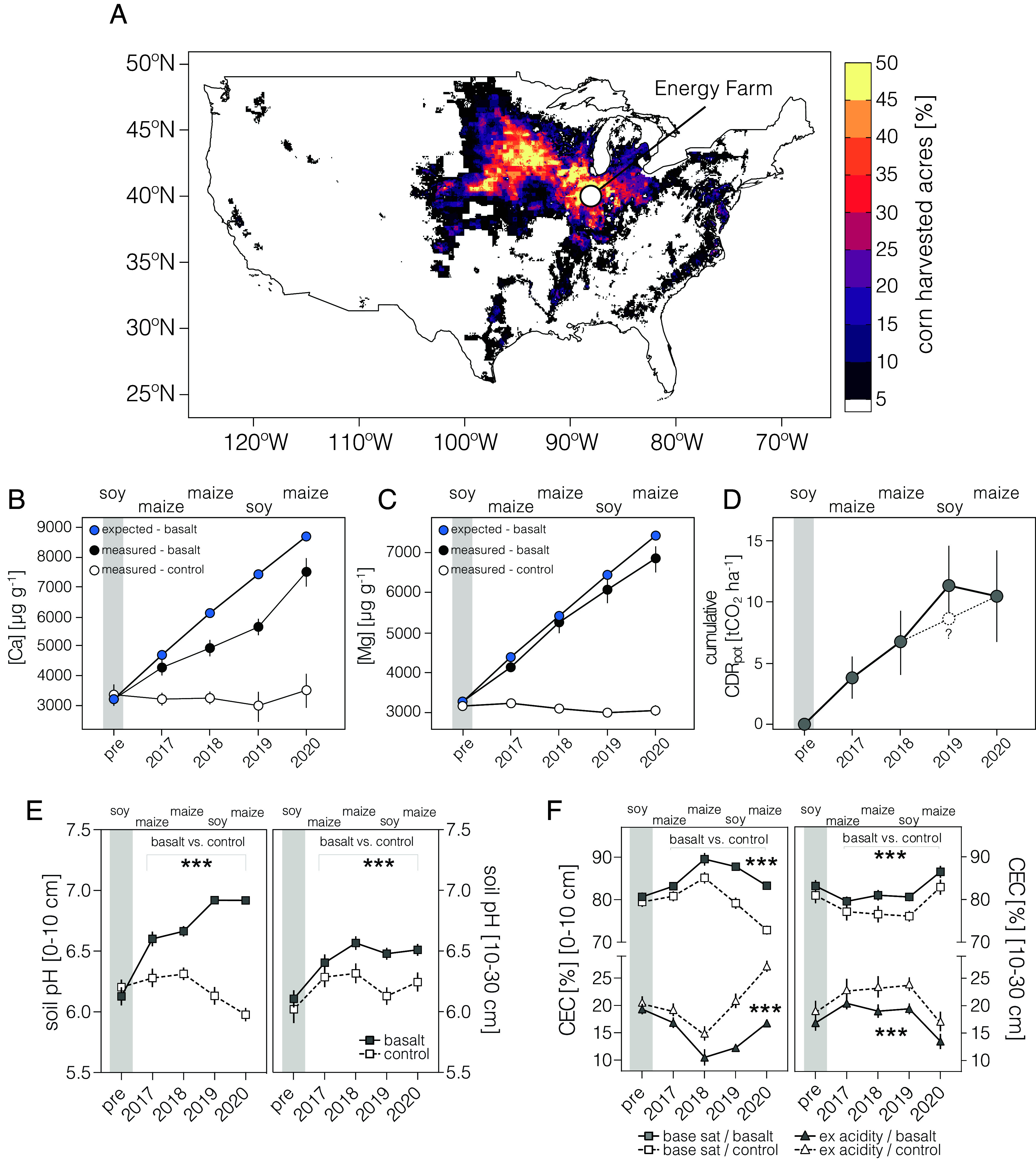
Field trial CDR potential and soil biogeochemistry changes in response to EW. (*A*) Harvested corn acres across the US Corn Belt, shown as regional percent area (12). The field trial site is shown with an open circle. (*B*) Time-series of expected and observed soil calcium (Ca), (*C*) Mg cation concentrations in treated and control plots. (*D*) Cumulative CDR potential (CDR_pot_) over our 4-y trial in a Midwestern corn–soy rotation. Results show mean CDR_pot_ across four sample blocks. The gray bar shows the pretreatment period (pre). The symbol “?” denotes a typical CDR_pot_ rate for maize in 2019. (*E*) Soil pH increased significantly with EW at 0 to 10 cm and 10 to 30 cm depths. (*F*) Soil CEC increases with EW while exchangeable acidity decreases at 0 to 10 cm and 10 to 30 cm depths. Error bars are ± SEM. Statistical results shown for repeated measures two-way ANOVAs with basalt vs. control as the main factor, asterisks indicate significant difference (**P < 0.01, ***P < 0.001).

The United States (US) Corn Belt represents 70 million hectares of intensively managed agricultural land cultivating major fuel, food, and feed row crops. We report detailed results and analysis from a large-scale replicated EW field trial undertaken over 4 y (2016 to 2020) across a maize (*Zea mays* L.) and soybean (*Glycine max* L.) rotation on an experimental farm in Corn Belt (Fig. 1; 40°30′N, 88°11′W) (Fig. 1*A*). In our field study, we applied crushed basalt annually for 4 y (at a rate of 50 tons per hectare) to a large field (3.8 ha) and multiple (*n* = 4) smaller treatment plots and compared the results to control plots with the same size and replication (*SI Appendix*, Fig. S1) (11). By incorporating large and small plots, our field trial design addresses the performance of EW across spatially heterogeneous soils and allows statistical assessment of this technology in agronomic practice.

For the purposes of this work, the loss (weathering) of major divalent cations (Ca^2+^ and Mg^2+^) from the applied basalt feedstock is assumed to be charge balanced by the formation of bicarbonate ions (2, 4, 5, 9). This defines the time-integrated potential for atmospheric CDR (CDR_pot_) by EW. It allows quantification of the cumulative effects of multiple applications of basalt on CDR_pot_, as an essential first step in measuring the ultimate permanent CDR storage. We quantify weathering mass loss of major divalent cations from the added basalt grains using straightforward cation accounting in soils relative to immobile trace element concentration (e.g., refs. 13 and 14). Temporary (reversible) retention of weathered cations on soil exchangeable sites prior to drainage discharge (15–17), and downstream changes in CDR efficiency (18), are not directly accounted for. This soil-based technique complements conventional approaches to estimating weathering rates based on detailed geochemical analyses of catchment drainage waters (e.g., ref. 19). However, soil analysis overcomes the fundamental challenges of applying drainage chemistry approaches in Midwestern croplands including a high and variable background pool of base cations in bulk soil, high background alkalinity fluxes, and variable hydrology. Furthermore, farms routinely analyze soils for pH and nutrient management. Consequently, estimating cation loss with a soil-based technique could be integrated for monitoring, reporting, and verification (MRV) of CDR_pot_ by EW on working farms to facilitate rapid upscaling.

Here, we report in situ soil EW rates from our large-scale field trial over 4 y. We also assess how EW affects key soil health metrics over time including pH, nitrogen availability, release of phosphorus (P) and potassium (K), and the pH-dependent availability of other nutrients important for healthy crops, including molybdenum (Mo) and silicon (Si). We report maize and soybean yield responses to EW over multiple years, compare the response to a traditional liming treatment (limestone amendment), and assess root transcriptional responses of both crops to provide mechanistic insights explaining observed yield responses. Our study represents a long-term EW field trial with basalt on an experimental farm, integrating evidence across geochemistry, soil science, and molecular genetics, to build a comprehensive picture of the operational performance of EW for the major agronomic ecosystem in the Midwest.

## Results and Discussion

### EW and CDR Potential.

We report average in situ loss of 32 ± 13% (SEM) for Ca^2+^ (*P* < 0.05) and 12 ± 10% for Mg^2+^ (*P* > 0.05) cations from basalt in treated soils at 0 to 10 cm depth relative to controls across a conventional corn–soy rotation under field conditions over 4 y (Fig. 1 *B* and *C* and *SI Appendix*, Figs. S2 and S3). The combined mass loss of these major divalent cations (16 ± 6%) was significant (*P* < 0.05). Preferential loss of Ca^2+^ reflects the higher abundance of faster weathering Ca-minerals (e.g., ferroactinolite) compared to slow weathering Mg minerals (e.g., chlorite) in the applied basalt feedstock (20). The resulting cumulative CDR_pot_ trend increases from 3.8 t CO_2_ ha^−1^ in the first year of treatment to ~10.5 t CO_2_ ha^−1^ after four annual rock dust applications (Fig. 1*D*). The CDR_pot_ curve represents the rates expected for weathered cations eventually exported from the soil profile as charge equivalents of bicarbonate. The average annual CDR_pot_ rate from these trials is comparable to a previous single year (2020) carbon removal estimate from the same field study (11). It is higher than rates of soil organic carbon sequestration linked to shifts in agricultural practices (e.g., ref. 21). The CDR_pot_ rate was highest during the soybean year in our rotation (2019), which is consistent with highly localized acidification by the rhizosphere of N_2_-fixing legume roots increasing weathering (22, 23). Consequently, the apparent flattening of the CDR_pot_ curve is likely a transient feature due a change in crop functional type. An average rate for maize instead of soybean in 2019, for example, would be consistent with a cumulative linear rise in CDR_pot_ (Fig. 1*D*).

Considerable scope exists to realize higher rates of CDR in the field with EW. Field-based CDR_pot_ rates of ~3.4 t CO_2_ ha^−1^ y^−1^ were achieved with application of a crushed metabasalt characterized by a grain size *p*80 of 267 µm (defined as 80% of the particles having a diameter less than or equal to this specified particle size), that contained a low proportion (~11% mass) of fast-weathering minerals (20). This rate of CDR compares to a theoretical maximum CDR potential of 16.2 t CO_2_ ha^−1^ per 50 t rock ha^−1^, based on weathering of Ca^2+^ and Mg^2+^ in the applied basalt (Dataset S1 gives feedstock composition). Optimization of rock dust application rates, use of finer-grained material, and selection of basalts with faster weathering mineralogies than the by-product metabasalt used here, hold promise for achieving substantially higher CDR rates (20).

The CDR_pot_ curve (Fig. 1*D*) is based on cation accounting in soils. Because of the removal of cations from the field in harvested grain (other plant biomass remains in the field), we measured base cation uptake into maize and soybean grains. A small, statistically significant, increase occurred in grains but only accounts for 0.3% of the total cation release from basalt. There was no significant increase in measured peak biomass cation content (*SI Appendix*, Figs. S4 and S5).

Soil pH increased (*P* < 0.001) in the surface layer (0 to 10 cm) and deeper in the profile (10 to 30 cm) with EW over 4 y, thereby preventing soil acidification that occurs regularly from nitrogen fertilization, as seen in control plot soils (Fig. 1*E* and *SI Appendix*, Fig. S6). Thus, observed soil profile pH responses over multiple years demonstrate a primary effect of EW (2, 3). Buffering of soil pH is evident in the near-neutral range in the treated plots, consistent with the protonation of soil organic and mineral surfaces (24). This offers evidence on the time scale of the trial that pH increase is not necessarily a limiting factor for future repeat applications of basalt. Further evidence of EW is shown by treated soils undergoing increases (*P* < 0.001) in the base cation saturation [as a percentage of cation exchange capacity (CEC)] of bulk soil compared to control soils, as Ca^2+^ and Mg^2+^ ions released from basalt weathering replaced exchangeable acidity (Fig. 1*F* and *SI Appendix*, Fig. S6) over time.

The increase in soil pH may favor the possible formation of carbonate mineral phases. However, we found no detectable increases in total inorganic carbon for a representative subset of bulk soil samples (0 to 10 cm and 10 to 30 cm depths, across blocks and sampling dates, including control and treated samples), with all measurements being below detection limit of 0.1% wt C. Thus, there is no evidence for pedogenic carbonate formation in topsoil as a significant sink for inorganic carbon, in agreement with findings from mesocosm trials with basalt amended acidic agricultural soil (25).

Soil pH responses highlight the ability of basalt to potentially replace agricultural limestone application while simultaneously capturing carbon and lowering other GHG emissions (3, 26). Limestone is commonly used to manage levels of soil acidity that often limit yields throughout the Corn Belt (27). This replacement offers a substantial benefit, given soil pH regulation with limestone typically costs farmers upward of ~$25 t^−1^ (28) and, at the field scale, can emit millions of tons of CO_2_ annually (27, 29). At the catchment scale acidity consumed by limestone weathering (or strong acid weathering of basalt) can reduce the export of acidity from soil drainage waters and thus lower CO_2_ evasion from river systems, albeit on variable timescales after rock application (30). The CO_2_ evasion from surface waters in the United States is substantial (97 ± 32 Mt CO_2_ y^−1^) (31), suggesting this process could have a non-negligible impact on catchment net carbon balance. In our study, strong acid weathering due to N-fertilizers reduces annual CDR_pot_ rates, with the most conservative accounting, by 5.2% at the field scale.

Our study aimed to provide empirical constraints on the dissolution rates of crushed basalt feedstock in a typical Corn Belt setting. In acidic soils, such as at the Energy Farm, the transport flux of cations and dissolved inorganic carbon species in drainage water can significantly lag the rate of EW in the soil. The lag is due to reversible retention of dissolution products on reactive mineral phases and organic surfaces (15–17) and does not affect the overall amount of CDR that will occur. However, it retards the movement of chemical tracers of weathering through the system, with a delay exceeding soil water residence times (32). This phenomenon, often discussed in terms of a solute transport retardation factor, is a well-established concept within soil science (15, 33). Time-lags in the export of weathered cations and bicarbonate alkalinity from treated soils likely explain the lack of near-term evidence for strong EW signals in stream water in small catchment oil palm field trials in Malaysia (19).

Our soil-based analysis has the advantage of providing a time-integrated quantification of the loss of reactive mineral cations in forward weathering reactions that drive carbon removal in the field (14). It avoids the need to quantify the spatial and temporal variability in water movement that transports soluble EW CDR reaction products (alkalinity, cations, and dissolved inorganic carbon). In situ measurement of bicarbonate alkalinity in soil pore waters provides a confirmatory snapshot of CDR following EW in field trials with maize in California (34). Importantly, however, determining cumulative CRD at any point in time currently requires solid-soil analysis with an appropriately detailed soil sampling regime to capture farm-scale spatial heterogeneity.

Reproducible, empirical quantification of tons of CDR_pot_ per hectare per year based in soil cation accounting provides proof of concept for EW with basalt. These results generally support model assessment of EW and CDR capacity on farmland with basalts of differing mineral chemistry and repeated annual applications of crushed rock over consecutive years (4, 5). Our assessment of CDR at this field site suggests EW can provide a significant offset against the greenhouse gas emissions from conventional agriculture in the region (11). By demonstrating attribution of CO_2_ removal potential under field conditions, we advance the feasibility of EW for the agroecosystem that is most central to US agricultural production and global food security. Our results also represent an initial step to develop robust MRV of CDR through EW with basalt on farmland. However, further work is required to assess the fate of captured carbon during transport by rivers (18) and its release in the coastal oceans (8, 9) as important steps along the pathway to marine sequestration on a timescale exceeding 10,000 y.

### Soil Fertility.

**Fig. 2.**
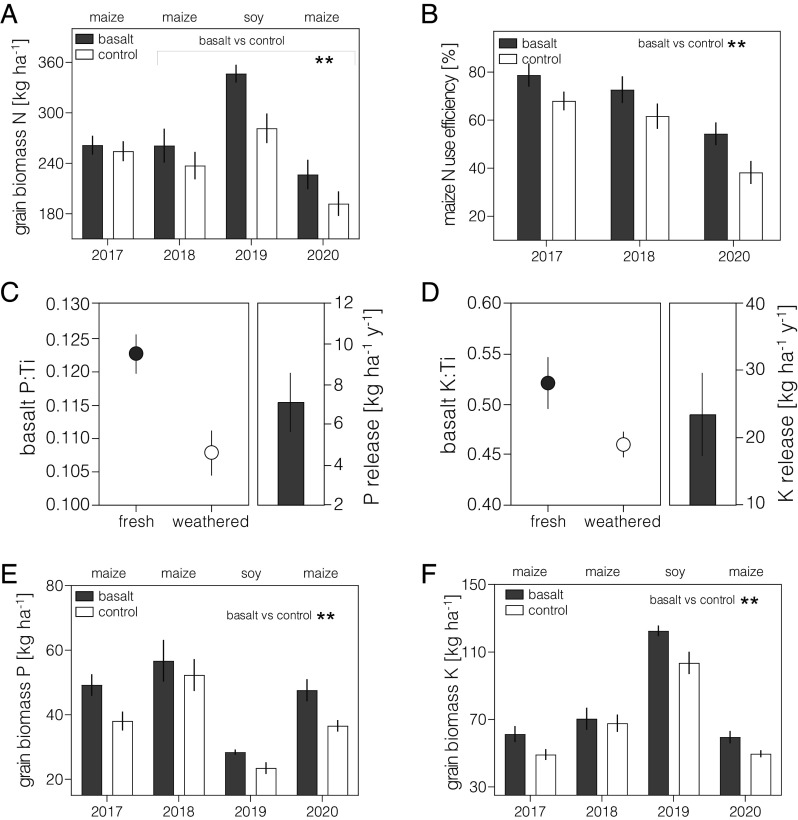
Field trial soil and crop biogeochemical responses to EW. (*A*) Total grain biomass nitrogen (N). (*B*) Plant NUE. (*C*) Measured P to Ti ratios in fresh and weathered basalt and calculated release of pf P. (*D*) Measured K to Ti ratios in fresh and weathered basalt and calculated release of P and K on a mass per unit area basis. (*E*) Total grain P and (*F*) K both increased significantly with EW. Error bars are ± SEM. Statistical results shown for repeated measures two-way ANOVAs with basalt vs. control as the main factor, asterisks indicate significant difference (**P < 0.01, ***P < 0.001).

Improved soil fertility following mineral nutrient release, and increased nutrient availability with the rise in pH as basalt weathers, are important potential cobenefits of EW alongside reversal of soil acidification that have not yet been quantified for EW field trials (3, 6). We focus on nitrogen (N), P, and K, the primary nutrients supplied by expensive chemical fertilizers to lift Corn Belt yields, and Mo and Si as key subsidiary nutrients (35, 36) essential for healthy crops and yields (Fig. 2).

We found significant (*P* < 0.01) increases in the total grain biomass N of maize and soybean in response to EW treatments (2017 to 2020). Increased grain N stocks in maize with EW arise largely from greater remobilization from vegetative biomass N-pools (*SI Appendix*, Figs. S7 and S8), leading to increased (*P* < 0.05) nitrogen use efficiency (Fig. 2 *A* and *B*). In contrast, increased N-supply in soybean grain results from increased soil uptake and N_2_ fixation with EW (*SI Appendix*, Fig. S9).

Mo is a key cofactor for nitrate reductase in biomass, the enzyme catalyzing conversion of nitrate to nitrite, which plants ultimately convert to ammonium for amino acid biosynthesis, and for symbiotic N_2_-fixation (35). We show that the increased soil pH driven by EW (Fig. 1*E*) facilitates mobilization of molybdate anions in soil porewaters in agreement with established desorption kinetics (37), increasing biomass Mo stocks in soybean and maize (*SI Appendix*, Fig. S10) and improving nitrogen assimilation and NUE (nitrogen-use efficiency).

Release of P and K from basalt can be an important additional benefit of EW, saving costs by reducing reliance on expensive fertilizers (5, 38) but this possibility remains to be quantified in EW field trials. We therefore determined the release of P and K in soil from basalt by EW by X-ray fluorescence (XRF) analysis of magnetically extracted basalt grains from soils in 2020 for comparison with unweathered grains. We show significant reductions in the P/Ti and K/Ti ratios in weathered compared to fresh basalt and calculate the annual application of crushed basalt (50 t ha^−1^) released an average of at least 7 kg P ha^−1^ y^−1^ and 23 kg K ha^−1^ y^−1^ by EW over 4 y (Fig. 2 *C* and *D*). These represent 20 to 30% and 25 to 40% of typical P and K fertilizer application rates, respectively, for soybean and maize in the Mid-west. However, our rates of release by EW are likely lower estimates, given not all basalt grains contain iron-bearing magnetic minerals and fine grains will have undergone complete dissolution. These results highlight the potential for EW to reduce the amount of expensive P and K fertilizers [urea phosphate, $890 t^−1^; diammonium phosphate, $938 t^−1^, potash $862 t^−1^], with important economic and environmental savings for farmers (5).

We sought independent evidence that the crops responded to increased P and K release by measuring stocks of both elements in grains of crops from EW-treated and control plots. We show total mass of P and K in the grain of maize and soybean increased significantly (*P* < 0.01) with EW (Fig. 2 *E* and *F*), with basalt weathering providing the additional P. Increased soybean K acquisition is matched by release from slow weathering of K-bearing silicates, whereas for maize grain K is attributable to reallocation of K pools from vegetative biomass to the grain.

Si is beneficial for crop productivity and resistance to stress (39, 40). Accessibility of biologically available Si declines with a rise in pH as it becomes increasingly retained on mineral surfaces (36, 37). However there was no significant (*P* > 0.05) change in peak biomass Si of maize and soybean in response to EW over 4 y, despite decreasing Si availability with a rise in soil pH (Fig. 2*E*), (*SI Appendix*, Figs. S11 and S12). This indicates that Si release by basalt weathering compensated for reduced availability as soil pH increased. Subsequent pH-dependent soil analyses of adsorbed silica species showed an immobilized reservoir of Si released via EW as soil pH falls. Thus, EW can help redress depletion of biologically available Si pools caused by removal of biomass as part of current US farming practices which can limit yields (36), highlighting another advantage of EW relative to liming.

### Crop Root Transcriptional Responses to EW.

**Fig. 3.**
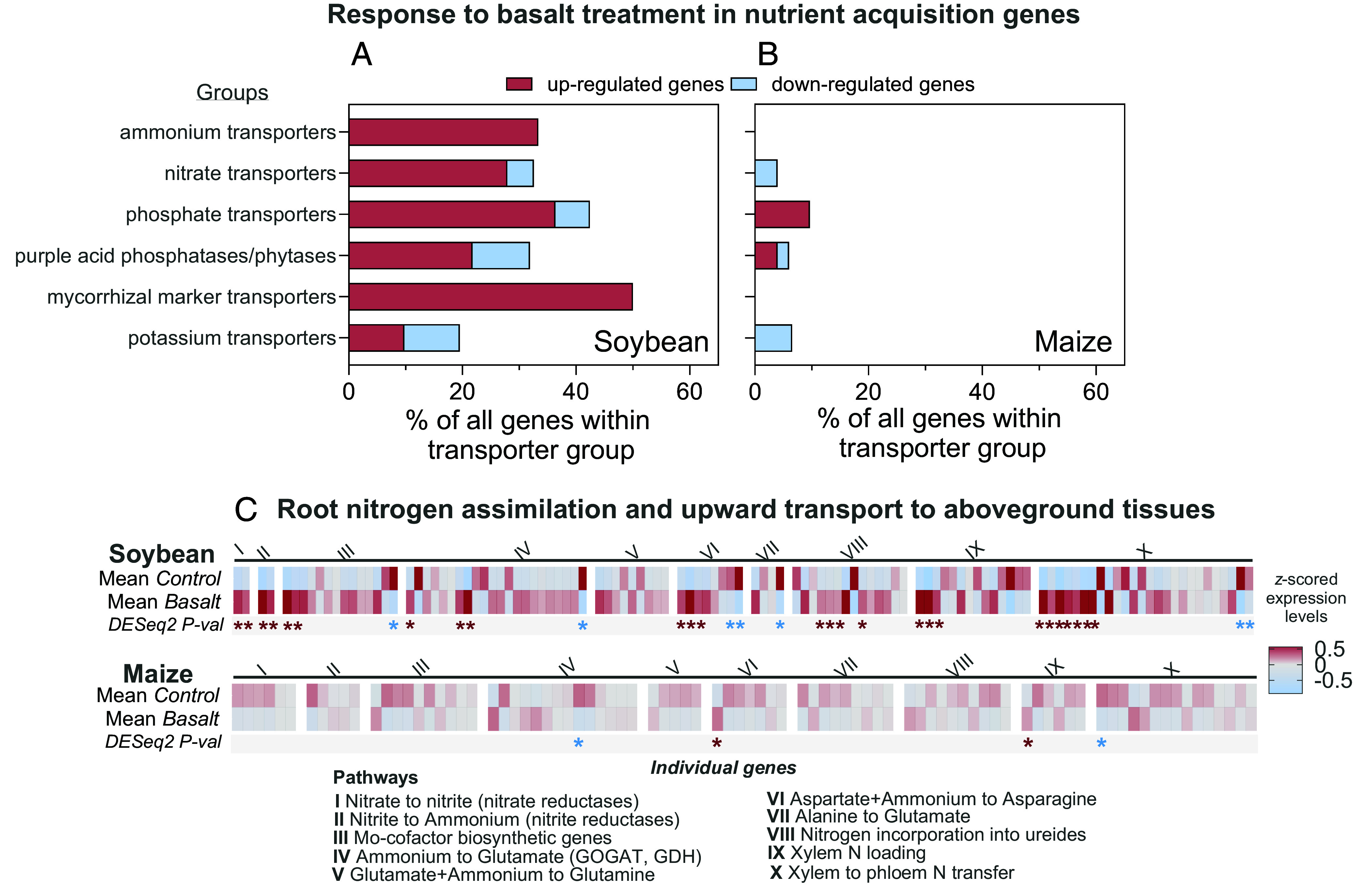
Root transcriptional responses of crops to EW under field conditions. (*A*) Soybean and (*B*) maize root nutrient transporter and acid phosphatase transcriptional responses to EW. Responses are expressed as the percentage of significantly up- and down-regulated transporter genes (differential expression DESeq2 tests, *P* < 0.05) within all genes in a transporter group in response to EW (i.e., between basalt and control plants). (*C*) Heatmap of z-score normalized root expression levels of genes involved in different pathways (labeled I to X) of root nitrogen assimilatory metabolism and long-distance upward transport. The heatmap shows that a whole suite of genes is differentially expressed (DESeq2, *P* < 0.05; blue asterisks denote down-regulated and red asterisks up-regulated genes) in response to basalt in soybean, but not maize.

We quantified soybean (2019) and maize (2020) root gene transcription changes for plants sampled from across blocks of the field trials at comparable reproductive developmental stages to gain mechanistic insight into the regulation of nutrient (N, P, and K) uptake responses to EW during grain filling. Transcriptional profiling of the resulting 47 high-quality root RNA-Seq libraries (*SI Appendix*, Fig. S13), focusing on inorganic ion transporter genes, showed a significant proportion of transporters for ammonium and nitrate, phosphate, and K in soybean roots responded to basalt (Fig. 3*A*). Upregulation of transporter genes, consistent with the specific requirements of soybean for increased soil uptake of N, P, and K during grain filling dominate changes in root gene expression. Maize roots showed the strongest upregulation response in the group of phosphate transporter genes with EW, consistent with its requirement for soil P during grain filling (*SI Appendix*, Fig. S7), and downregulation of K and N-transporter genes, as expected with reallocation of K and N pools from vegetative biomass to grain (Fig. 3*B*). Additionally, genes expressing acid phosphatases and phytases were significantly up-regulated with EW in both soybean and maize. These responses are consistent with increasing P release by enzymatic breakdown of organic matter (41) to assist in P uptake during grain filling. Symbiotic mycorrhizal fungal-to-root transporters of P and N (42–44) were up-regulated in soybean, but not in maize, where high fertilizer applications likely lead to loss of mycorrhizal fungal partners.

Root transcriptome analyses further support the role of improved Mo availability in enhancing soybean N nutrition (35) with genes involved in Mo cofactor biosynthesis up-regulated in response to EW, together with the genes involved in nitrate assimilation including nitrate and nitrite reductases (Fig. 3*C*). Transcriptomes showed upregulation of genes involved in conversion of root-acquired inorganic N to organic forms (pathways I to VIII) and long-distance transport genes exporting root N to aboveground soybean biomass (pathways IX and X) (45) (Fig. 3*C*). However, in maize where N remobilization from vegetative biomass pools was sufficient, we saw no upregulation of this suite of genes. Overall, transcriptional reprogramming of root nutrient transporters in both crops balanced grain biomass requirements in response to EW. This provides genomic evidence independently supporting the observed changes in plant nutrient budgets.

### Crop Yield Responses.

**Fig. 4.**
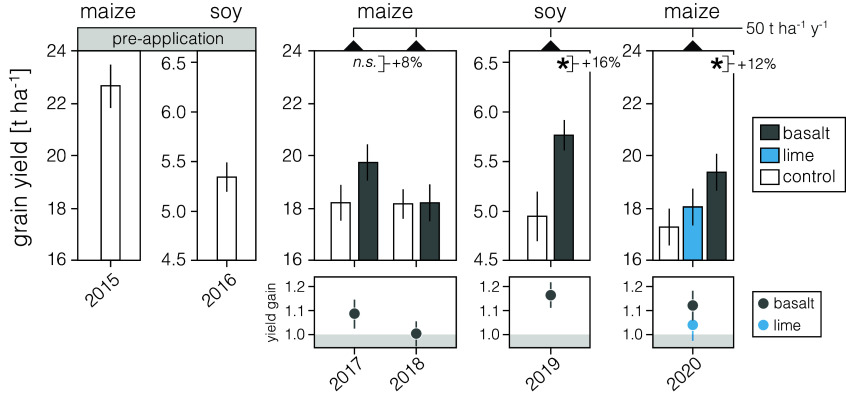
Grain yield responses of food and energy crops to EW and liming under field conditions. Results are averaged across hand-harvesting of large and small blocks. Error bars are ± SEM. Statistical results shown for two-way ANOVAs with basalt vs. control as the main factor, asterisks indicate significant difference (*P < 0.05).

Grain yields determined by replicate hand harvesting from the small and large blocks of the field trials increased significantly with EW for maize (a C_4_ photosynthetic crop) by 12% (2020) and soybean (a C_3_ photosynthetic, N_2_-fixing crop) by 16% (2019) (Fig. 4 and *SI Appendix*, Fig. S14). Yield increases for soybean with EW are comparable to those for bioengineered soybean (46) and soybean grown under well-watered conditions with elevated CO_2_; well-watered maize in contrast to EW shows no yield enhancement with elevated CO_2_ (47).

Observed yield enhancements are broadly consistent with those from EW mesocosm trials using crushed basalt (25) and EW field plots and experiments using wollastonite (48). We attribute yield gains in both crops to the combined effects of increased nutrient supply as basalt minerals dissolve, increased nutrient availability with rising soil pH (Fig. 2), and greater root uptake of mineral elements (Fig. 3). This is supported by the lack of a significant maize yield response to liming in 2020 (Fig. 4), suggesting basalt provided inorganic nutrients in addition to raising soil pH. Significant yield increases in maize were only observed in those years of the rotation following soybean, suggesting an EW-nitrogen interaction (49). Extrapolated across the Corn Belt region, EW-related yield increases translate to ~$7-11 billion for maize, and $10 billion for soybean at current prices, indicating EW deployment could deliver substantial economic benefits for US agriculture through yield increases alone.

Yield enhancements with EW were achieved with significantly (*P* < 0.05) increased key micro- and macronutrient concentrations [including K, magnesium (Mg), manganese, P, and zinc], thus improving or maintaining crop nutritional status (*SI Appendix*, Fig. S15). We observed no significant increase in the content of trace metals in grains of maize or soybean (*SI Appendix*, Fig. S16), soil pore water or soil exchangeable pools after four annual EW treatments relative to controls (*SI Appendix*, Figs. S17 and S18). Mo, essential for plant N metabolism increased approximately fourfold with EW over this time and copper, an essential micronutrient for photosynthesis at these levels, increased around 35%. Overall, based on 2,000 measurements of 12 trace metals at two soil depths (0 to 10 cm and 10 to 30 cm) over 4 y of repeat basalt application, these data help alleviate concerns over possible accumulation of bioavailable metals in soils with basalt-based EW (50).

## Conclusion

We show with a large-scale replicated field trial over 4 y that significant annual divalent cation loss from crushed basalt applied to soils drives long-term CDR_pot_ under field conditions across a conventional corn–soy rotation in the US Corn Belt. Our field study demonstrates the use of in situ soil EW measurements that could potentially form part of a robust MRV toolkit for quantifying rates of basalt weathering and time-integrated CDR. Such empirical quantification of potential CO_2_ removal rates in the field is an essential first step for determining ultimate permanent CDR storage in the oceans and a prerequisite for MRV to facilitate wide-scale adoption of EW. We also quantified major agronomic benefits of EW for this dominant agroecosystem, including increased crop yields (maize and soybean) via improved soil fertility and changes in root gene expressions, without adverse environmental impacts for plants and soils. Collectively, our evidence supports EW with basalt as a promising strategic management option for atmospheric CDR, deployable with existing agricultural practices and equipment. These findings highlight the capacity of EW to simultaneously augment food security and soil health while delivering CDR and generating revenue for critical agricultural regions.

## Materials and Methods

### Energy Farm Site, EW Operations, and Field Collection.

Research was conducted at the University of Illinois Energy Farm (40°3′46″N, 88°11′46″W), south of Urbana, Illinois, in 2016 to 2020 (see refs. 11 and 51 for details). The EW experimental trial design consisted of four 0.7 ha blocks each containing four 10 × 10 m subplots, with 10 × 16 m buffer zones separating basalt and control subplots, and two 3.8 ha large fields overall (*SI Appendix*, Fig. S1). In a randomized block design, Blue Ridge basalt rock dust (Speciality Granules, Blue Ridge Summit, PA) (20) (Dataset S1) was applied (50 t ha^−1^) to two of the subplots within each the four 0.7 ha blocks and one entire 3.8 ha field to create eight 100 m^2^ treated subplots and one 3.8 ha treated plot, with the other eight 100 m^2^ subplots and 3.8 ha plot serving as controls (11). Given that subplots within a block are not true replicates, both subplots of the same treatment within a plot were sampled independently and data were then averaged together for a total experimental design of *n* = 5. All plots were managed in a maize (*Zea mays* L.)-maize-soybean (*Glycine max* L.) rotation typical of the region. Nitrogen fertilizer was applied annually prior to maize planting as 28% urea ammonium nitrate at 202 kg N ha^−1^; fertilizer was not applied to soybean. Rock dust was applied annually in November 2016, 2017, 2018, and 2019 using conventional lime spreading equipment, and subsequently chisel plowed into the soil to a depth of ~18 cm within 24 h.

We added a limed plot treatment to increase soil pH for comparison with the basalt treatment. Within each of the four 0.7 ha plots, two additional 10 × 10 m subplots were established, and granular lime added by hand with a pushed broadcast spreader at a rate of 6.7 t ha^−1^ (3 US ton/acre) on April 22, 2020 and cultivated into the soil during planting activities. These plots were sampled and treated statistically the same as the control and basalt subplots established in 2016 (8 total subplots per treatment, *n* = 4 for all comparisons with limed plots). Measurements were collected as for all other plots.

Above- and below-ground biomass was collected each year at peak biomass (determined as described in refs. 11 and 51) and yield collected immediately prior to plot scale harvest. A randomly placed 0.75 × 0.75 m quadrat was used to collect above-ground biomass, and the materials were sorted and processed as described by Kantola et al. (11), while grain only was collected for yield prior to harvest. This quadrat size ensures harvesting at least one row each time regardless of orientation. Within each subplot, two locations were harvested, resulting in a total of four locations per treatment within a block. Within each large plot, four locations were harvested. Values from small and large plots were used for statistical comparisons at the block level (*n* = 5). Within each quadrat placed to sample peak biomass, three soil cores, 30 cm depth, 5.1 cm diameter, were taken with a slide hammer (AMS Inc., American Falls Idaho, USA), divided into 0 to 10 and 10 to 30 cm depth increments and samples for each depth pooled and processed as described previously (11).

### Plant Tissue Chemical Analysis.

Homogenized plant powders were weighted to 200 mg in tubes followed by sequential addition of 3 mL of ultrapure concentrated HNO_3_ (Primar grade, Fisher Scientific, UK), 3 mL Milli-Q H_2_O, and 3 mL H_2_O_2_. Acid digestion was carried out on a microwave (Anton Paar Multiwave). Tissue concentrations of Si were measured via precalibrated XRF (Thermo Scientific Niton XL3t GOLDD+) analysis of pelletized plant powders under helium atmosphere (52). Dried and ground plant tissues were analyzed for C and N content on an elemental analyzer as described by Kantola et al. (11).

Tissue concentrations (in mg nutrient kg^−1^ dry biomass) were corrected for background matrix effects using blank values and were subsequently multiplied with their respective harvested dry biomass weight (in kg dry biomass ha^−1^) to obtain a nutrient pool figure (kg nutrient ha^−1^). Total peak biomass nutrient pool estimates were based on the sum of root, stem, leaf, and floral nutrient pools collected at August peak harvest. Vegetative peak biomass nutrient pool figures were based on the sum of root, stem, and leaf pools for the given nutrient. Total grain biomass pools were derived as the product of grain tissue concentration and grain dry biomass. Replication for each of the two treatment (treated and control) was *n* = 10 samples (2 subplots × 4 small sites = 8 and 2 samples from a large plot) per pool per annum to a total of 80 measurements per nutrient pool across the 4-y trial period. Nitrogen isotope analysis (δ^15^N) of plant samples was undertaken by Iso-Analytical Ltd., Crewe, UK, using Elemental Analysis–Isotope Ratio Mass Spectrometry. The reference material for plant samples was IA-R001 (wheat flour, δ^15^N AIR = 2.55‰).

### Soil Analyses.

Soil samples were treated with ammonium acetate to leach the non-mineral-bound exchangeable fraction and subsequently prepared for metal analysis using isotope dilution (ID-) ICP-MS (inductively coupled plasma mass spectrometry) at the Yale Metal Geochemistry Center (14). Soil pH was measured following previously described procedures (53), and soil exchangeable acidity was measured using a soil-buffer equilibration method (54) with substitution of 0.1M sodium phosphate buffer for the highly toxic Shoemaker-McLean-Pratt buffer reagent (55). Soil exchangeable cations were analyzed by extracting soils with a saturated salt solution (56). CEC was calculated as the sum of base cations and exchangeable acidity.

The availability of many soil nutrients depends on soil pH (56–58). As soil extractants are for specific pH values, making conclusions regarding resulting nutrient availability to plants under field conditions difficult. To resolve this, we reconstituted air-dried soil samples with ultrapure water thus bringing the soil sample to its native pH range. Reconstituted soil samples were briefly heated to boiling temperature (20 min) to allow for greater desorption to increase concentrations and improve ICP-MS detection. Sample filtrates and three blanks were acidified to 2% nitric acid, and their multielemental composition was analyzed using ICP-MS.

We control for pH to allow estimating soil Si pools at equivalent pH (59). For this, 10 g of air-dried soils were extracted with 25 mL 0.1M sodium phosphate buffer solutions set up at three different pH values—pH 6.0, pH 6.5, and pH 7.0. The resulting mixtures were left to equilibrate for 72 h at 4 °C. Samples were subsequently extracted using hot water bath as described in the previous section. A subsample of 2 samples of treated and 2 control soils were analyzed from pretreatment collection and 5-y posttreatment collection (*n* = 5 blocks for 2016 and 2021).

Nitrogen mineralization rates were calculated as the loss of soil N in the top 30 cm for each treatment at each block between 2016 and 2020 (*SI Appendix*, Fig. S8). These were averaged for treatment and converted to kg N loss ha^−1^ y^−1^ by multiplying the soil N loss (in %) with estimated amount of soil in top 30 cm and dividing by the number of years. Treatment-specific N-mineralization rates were used in the calculation of NUE and assumed to be constant throughout the 4-y trial period. NUE were calculated by the established methods (60) following formula:

$$\text{NUE}\%=\frac{N_{\text{grain}}{-N}_{\text{mineralization}}}{N_{\text{fertilizer}}}100,$$

where N_grain_ is the total grain biomass N pool (in kg ha^−1^), N_mineralization_ is the treatment-specific nitrogen mineralization rate, and N_fertilizer_ is the N fertilizer application rate.

### ICP-MS Procedures.

Multielement analysis of diluted solutions was undertaken by ICP-MS (Thermo-Fisher Scientific iCAP-Q; Thermo Fisher Scientific, Bremen, Germany). Multielement analysis of digested soil samples was undertaken at the Yale Geochemistry Center. Isotope dilution ICP-MS was used to reduce analytical error when measuring concentrations of Ca, Mg, and Ti (14, 61). We use an isotope spike cocktail, doped with minor isotopes of Mg, Ti, and Ca, as described in ref. 14. Estimate of the error on the spike determination are <0.1‰ based on replicate analysis. Samples were analyzed for ID-ICP-MD with a Thermo-Fisher Scientific Element High Resolution Magnetic Sector ICP-MS (Thermo-Fisher Scientific, Bremen, Germany) and a PerkinElmer NexION 5000 Multi-Quadropole ICP-MS (PerkinElmer, Waltham, MA, USA).

### EW and CDR_pot_ Calculations.

Cation loss from basalt in soils is the difference between the calculated loading based on basalt chemistry, application rate and the accumulation of an immobile trace element (titanium, Ti), and the residual cation content measured in soils for each year of the trial. We calculated the proportion of basalt (p_basalt_) in treated soil samples from 0 to 10 cm depth after four annual basalt applications of 50 t ha^−1^) (2020) using the accumulation of detrital Ti. This is resolvable because the basalt feedstock contains ~4 times more Ti than baseline soils (*SI Appendix*, Fig. S2) and was mixed through the entire 0 to 10 cm depth interval. p_basalt_ was calculated as:

[1] $$p_{\text{basalt}}=\frac{{\left[\text{Ti}\right]}_{\text{treated}}-{\left[\text{Ti}\right]}_{\text{soil}}}{{\left[\text{Ti}\right]}_{\text{basalt}}-{\left[\text{Ti}\right]}_{\text{soil}}}.$$

We solved Eq. **1** using the mean of 2020 basalt-treated soil samples across all blocks, [Ti]_treated_ = 3,417 ± 279 mg kg^−1^ (SD); the mean of 2016 pretreatment soil samples across blocks, [Ti]_soil_ = 2,693 ± 196 mg kg^−1^ (SD); and [Ti]_basalt_ = 10,899 ± 420 mg kg^−1^ (SD) to give p_basalt_ = 0.0881 ± 0.0417 (SD, *n* = 19), at 0 to 10 cm. Standard rules for error propagation were followed throughout.

The mass of basalt (m_basalt_) in treated soil samples was calculated as:

[2] $$m_{\text{basalt}}=\frac{m_{\text{soil}}\times p_{\text{basalt}}}{1-p_{\text{basalt}}}.$$

The mass of soil, per hectare, in the 0 to 10 cm depth (m_soil_) was calculated as:

[3] $$m_{\text{soil}}=v_{\text{soil}}\times q_{\text{soil}},$$

where volume of soil (v_soil,_ m^3^) is depth × area = 0.1 × 10,000 = 1,000 m^3^ and soil bulk density, q_soil_ = 1.2 t m^−3^ at the Energy Farm (62). Solving Eqs. **2** and **3** gave m_basalt_ ≈ 116 ± 5 t ha^−1^ (s.d.) (*n* = 19) at 0 to 10 cm depth. Therefore, we calculated that an average of 116 t of the 200 t of basalt applied after 4 years (58 ± 2.7%, s.d.) resided in the 0 to 10 cm depth horizon, indicating that the remaining 84 t (42 ± 2.7%) likely resided in the 10 to 30 cm depth horizon—as would be expected for the plowing regime used. This distribution was assumed in CDR_pot_ calculations throughout the 4-y trial period.

The expected concentration of Ca in treated soils for a given year, before any loss due to weathering, was calculated as:

[4] $${\left[\text{Ca}\right]}_{\text{expected}}={\left[\text{Ca}\right]}_{\text{basalt}}\times p_{\text{basalt}}+\left(1-p_{\text{basalt}}\right)\times {\left[\text{Ca}\right]}_{\text{soil}}.$$

The same expression was derived for [Mg]_expected_. We calculated weathered [Ca] as the difference between [Ca]_expected_ and [Ca]_observed_ (i.e., measured in samples from plots treated with basalt) for a given year:

[5] $${\left[\text{Ca}\right]}_{\text{weathered}}={\left[\text{Ca}\right]}_{\text{expected}}-{\left[\text{Ca}\right]}_{\text{observed}}.$$

The proportion of [Ca] lost from basalt because of weathering was derived from [Ca]_weathered_ as follows (and similarly for Mg):

[6.1] $$p_{\text{Ca}\_\text{weathered}}=\frac{{\left[\text{Ca}\right]}_{\text{weathered}}}{{\left[\text{Ca}\right]}_{\text{mix}\_\text{basalt}}},$$

where

[6.2] $${\left[\text{Ca}\right]}_{\text{mix}\_\text{basalt}}={\left[\text{Ca}\right]}_{\text{basalt}}\times p_{\text{basalt}}.$$

Eqs. **6.1** and **6.2** assume that the base cations lost via weathering originated from basalt rather than soil. This is supported by control plot data showing no significant changes in total soil Ca and Mg (Fig. 1 *C* and *D*). From the proportion of Ca and Mg lost from basalt, we calculated the potential CDR (CDR_pot_) as:

[7] $$\begin{array}{c} {\left[\text{CDR}\right]}_{\text{pot}}=\left(\frac{p_{\text{Ca}\_\text{weathered}}\times {\left[\text{Ca}\right]}_{\text{basalt}}\times 10^{-6}\times m_{\text{basalt\_applied}}}{A_{\text{Ca}}}\times 2+\frac{p_{\text{Mg}\_\text{weathered}}\times {\left[\text{Mg}\right]}_{\text{basalt}}\times 10^{-6}\times m_{\text{basalt\_applied}}}{A_{\text{Mg}}}\times 2\right)\times M_{{\text{CO}}_{2}}, \end{array}$$

where A_Ca_ and A_Mg_ are the atomic weights of Ca and Mg, respectively, M_CO2_ is the molecular weight of CO_2_, and m_basalt_applied_ is the total amount of basalt added by 2020. These calculations are based on our weathering measurements for 58% of the applied basalt in the 0 to 10 cm depth soils. Based on the significant increase in soil pH (Fig. 1*E*) and base saturation (Fig. 1*F*) at 10 to 30 cm depth, we assume the remaining 42% mixed into this depth horizon by chisel plowing underwent the same rate of cation loss. This 42% is likely a conservative estimate given higher soil *p*CO_2_ due to longer diffusion pathways for respired CO_2_ (63) and similar or lower pH (Fig. 1*E*) at depth, both which favor more rapid basalt weathering. Eq. **6** assumes that each mole of divalent cations weathered converts 2 moles of CO_2_ into HCO_3_^−^ based on the stoichiometry of the Urey reaction between silicate minerals and CO_2_ (64) (Dataset S6).

### Magnetic Extraction of Rock Grains and Rock XRF Analyses.

Basalt grains were recovered from treated soil samples using sequential magnetic extraction. For field weathered samples ~25 g of air-dried and ground basalt-treated soil samples (0 to 10 cm collected in August 2020) were placed in plastic weighing boats and strong neodymium magnets tightly covered in thin microscope lens paper run over the sample for 30 s. Basalt grains were placed into a fresh weighing boat by releasing the magnet over the fresh weighing boat and the procedure repeated three times. For a baseline, we used fresh unweathered basalt mixed with control soil samples that were brought to field moisture, dried and separated magnetically as described to account for bias. Magnet-extracted weathered basalt grains were measured for three samples per block (*n* = 5 blocks) and magnet-extracted fresh unweathered basalt grains (*n* = 5 blocks) were scanned using a Thermo Scientific XLt2 GOLDD XRF (20). XRF was calibrated using an international standard basalt sample BHVO-2. Each sample of basalt grains was scanned in Cu/Zn mining mode three times, and final values were obtained by averaging across the three measurements. Reproducibility for the measured elements was assessed as the SD between three separate measurements of the same sample giving: Ti (±1,115 ppm), K (±567 ppm), and P (±197 ppm) which represented 6.8%, 7.8%, and 10.6%, respectively, of mean values for these elements in the analyzed samples.

### Root RNA Extraction and Transcriptome Analyses.

Healthy green plants of average size were selected in the field from each treatment from different blocks. The roots of crop plants were collected from comparable developmental stages: soybean plants’ roots were collected at the end of July 2019, at the R4-R5 reproductive stage and roots collected from maize plants mid-August 2020, at the R3-R5 reproductive stage and roots were snap-frozen with liquid N_2_. Upon return to the laboratory, samples were stored in a −80 °C freezer prior to extraction. RNA was extracted from roots using the RNeasy® Plant Mini Kit (Qiagen) and purified to remove any contaminating DNA by using a RNase-Free DNase Set (Qiagen). The reaction was subsequently cleaned with RNA Clean and Concentrator kit columns (Zymo).

RNA integrity was assessed by gel electrophoresis with all samples exhibiting the characteristic 25S rRNA and 18S rRNA bands (*SI Appendix*, Fig. S13). To capture the coding transcriptome, cleaned total RNA samples were enriched for mRNA by the polyA tail-selection method using the Kapa RNA HyperPrep kit (Roche). mRNA library preparation was carried out with the TruSeq Stranded mRNA kit (Illumina). A total of 15 soybean root mRNA samples were sequenced, 8 for treated and 7 for control plants sampled across blocks, on one NovaSeq S1 lane (Illumina) using 2 x 150 nt paired-read chemistry. A total of 32 maize root mRNA samples were sequenced, 16 for treated and 16 for control plants sampled across blocks, on a S4 lane of the NovaSeq equipment using 2 × 150 bp paired-read chemistry. Sequencing reads were then uploaded onto the Galaxy Europe (https://usegalaxy.eu/) server (65). The cleaned paired-read libraries were aligned against their reference genome sequence (Gmax JGI Wm82.a2.v1 for soybean, and Zmays 493 APGv4 for maize) using HISAT2 (66). Unaligned reads were discarded and aligned reads were assembled using StringTie (67) with an average read length of 150 bp and a minimum assembled transcript length of 200 bp. Gene counts were normalized with the RUVseq tool (68). Samples were submitted for differential expression analyses through DESeq2 (69) using prefiltering of 1 read per sample and two factors—the primary factor treatment (basalt/control) and secondary factor—block from which samples are derived (Dataset S5). Detailed gene annotation for the particular reference genome version were obtained from Phytozome (70) (https://phytozome-next.jgi.doe.gov/).

### Economic Price of Yield Increases.

USDA data indicate that the Corn Belt is 50% corn and 50% soybean, which is broadly equivalent to US national average: 81 million acres corn/87 million acres soybean (71). We used a total Corn Belt area of 60 Mha giving 30 Mha (i.e., 74 million acres) for corn and soybean each (72). We used 2022 base yields of 173.3 bushel/acre (corn) and 49.5 bushel/acre (soybean) (73). Current (Feb. 2023) prices for corn: $6.44/bushel and soybean: $15.12/bushel (74). Over the course of the last 12 mo corn price varied by +26/−3% of current value, and soybean by 11/−12%. The value of increased corn/soy production with EW is calculated as corn/soybean area × corn/soybean base yield per area × fractional change in corn yield, e.g., for an 8.5% yield increase, the fractional increase in corn yield would be 0.085. We calculated the value of extra production as increased yield with EW × current price of corn/soybean.

## Supplementary Material

Appendix 01 (PDF)

Dataset S01 (XLSX)

Dataset S02 (XLSX)

Dataset S03 (XLSX)

Dataset S04 (XLSX)

Dataset S05 (XLSX)

Dataset S06 (XLSX)

## Data Availability

All study data are included in the article and/or supporting information.
