# Supplementary material for: Enhanced weathering in the US Corn Belt delivers carbon removal with agronomic benefits
Source: Proc Natl Acad Sci U S A. 2024 Feb 22;121(9):e2319436121. doi: 10.1073/pnas.2319436121 (PMC10907306; doi:10.1073/pnas.2319436121)
Supplement: Supplementary file 1 — Appendix 01 (PDF) [file pnas.2319436121.sapp.pdf]

Supporting Information

Enhanced weathering in the U.S. Corn Belt delivers carbon removal with agronomic benefits

David J. Beerling *et al.*

Correspondence to: d.j.beerling@sheffield.ac.uk

This PDF file includes:

Figs. S1 to S18

Data files:
Data File 1: Compositional data for Blue Ridge metabasalt feedstock
Data File 2: Compositional data for baseline soil samples (prior to basalt application)
Data File 3: Compositional data for soil samples taken at the Energy Farm EW trials
Data File 4: Muti-element ICP-MS limits of detection
Data File 5: Full gene listing and sample counts
Date File 6: Cation loss and CDR_{pot} calculations

Supplementary Figure listing

Fig. S1. Block design at the enhanced rock weathering experimental site at the Energy Farm, USA.

Fig. S2. Total elemental chemistry of pre-treatment (2016) soils and Blue Ridge basalt feedstock.

Fig. S3. Soil Ca and Mg pools across experimental blocks in response to EW.

Fig. S4. Pools of Ca and Mg in peak vegetative biomass across experimental blocks in response to EW.

Fig. S5. Pools of Ca and Mg in seed biomass across experimental blocks in response to EW.

Fig. S6. Soil pH and percentage exchangeable acidity across experimental blocks in response to EW.

Fig. S7. Minimum acquisition of N, P, and K during the grain filling stage, mid-August to mid-September.

Fig. S8. Changes in soil total N stocks and mineralization rates between 2016 and 2020.

Fig. S9. $\delta^{15}N$ of plant tissues for differentiation of symbiotically fixed vs. soil acquired N in soybean grains in response to EW.

Fig. S10. Soil and vegetation molybdenum budgets (Mo) over time.

Fig. S11. Soil and plant silicon dynamics following EW.

Fig. S12. Soil silicon (Si) in unbuffered hot water soil extracts at native soil pH.

Fig. S13. Spot checks of the integrity of total RNA extracts from plant roots.

Fig. S14. Crop yield responses to EW by trial blocks and year.

Fig. S15. Changes in grain concentration of macro- and micronutrients important for human nutrition with EW.

Fig. S16. Concentrations of trace metals in seeds from basalt-treated and control crops.

Fig. S17. Dynamics of trace metals in the soil pore water across depths.

Fig. S18. Dynamics of trace metals in the soil exchangeable pool across depths.

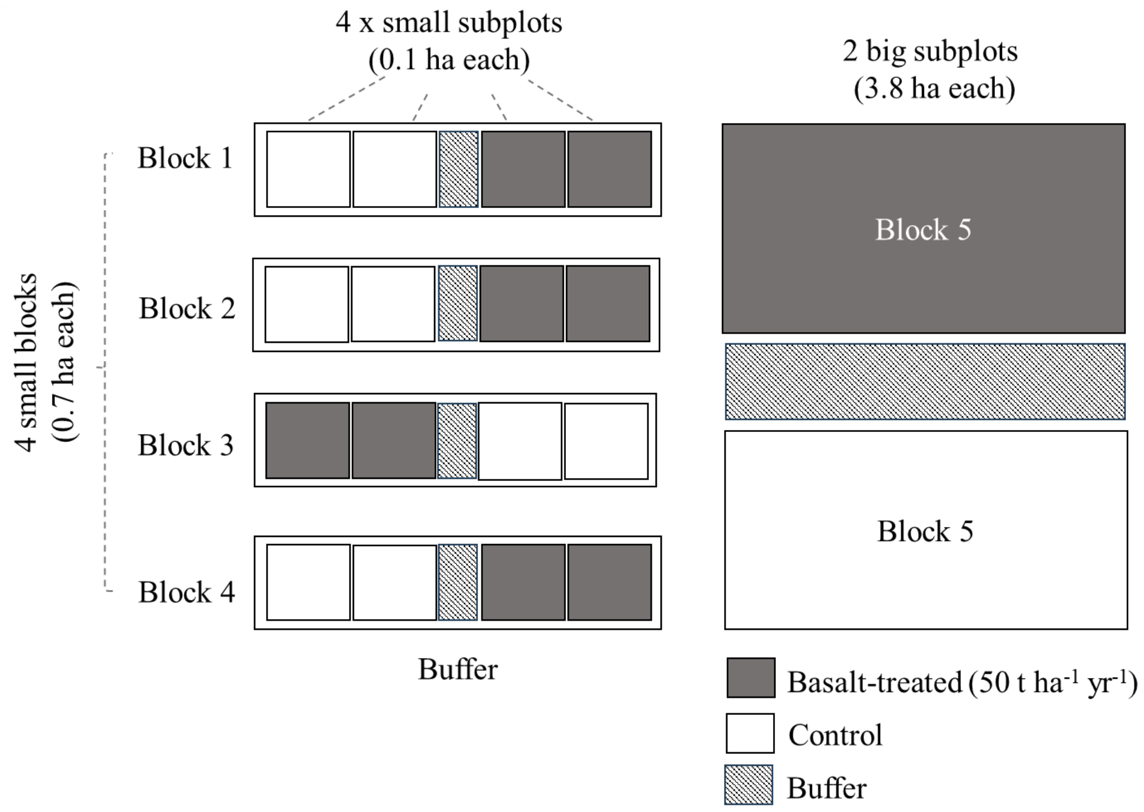

Fig. S1. Block design at the enhanced rock weathering experimental site at the Energy Farm, USA.

Each of four 0.7 ha blocks contains four 10 × 10 m subplots (×2 treated and ×2 control), with 10 × 30 m buffer zones separating basalt and control sub-plots. Additionally, there are block 5 (3.8 ha) large fields, one EW treated and one serving as a control (block 5). Not to scale.

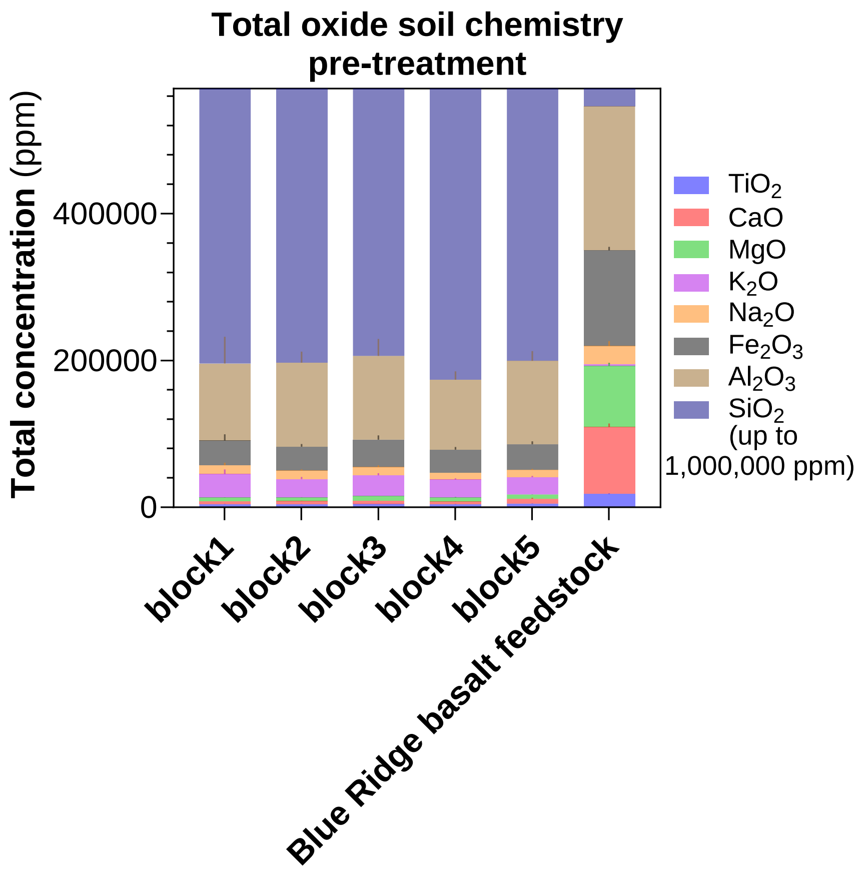

Fig. S2. Total elemental chemistry of pre-treatment (2016) soils and Blue Ridge basalt feedstock (Data Table 1).

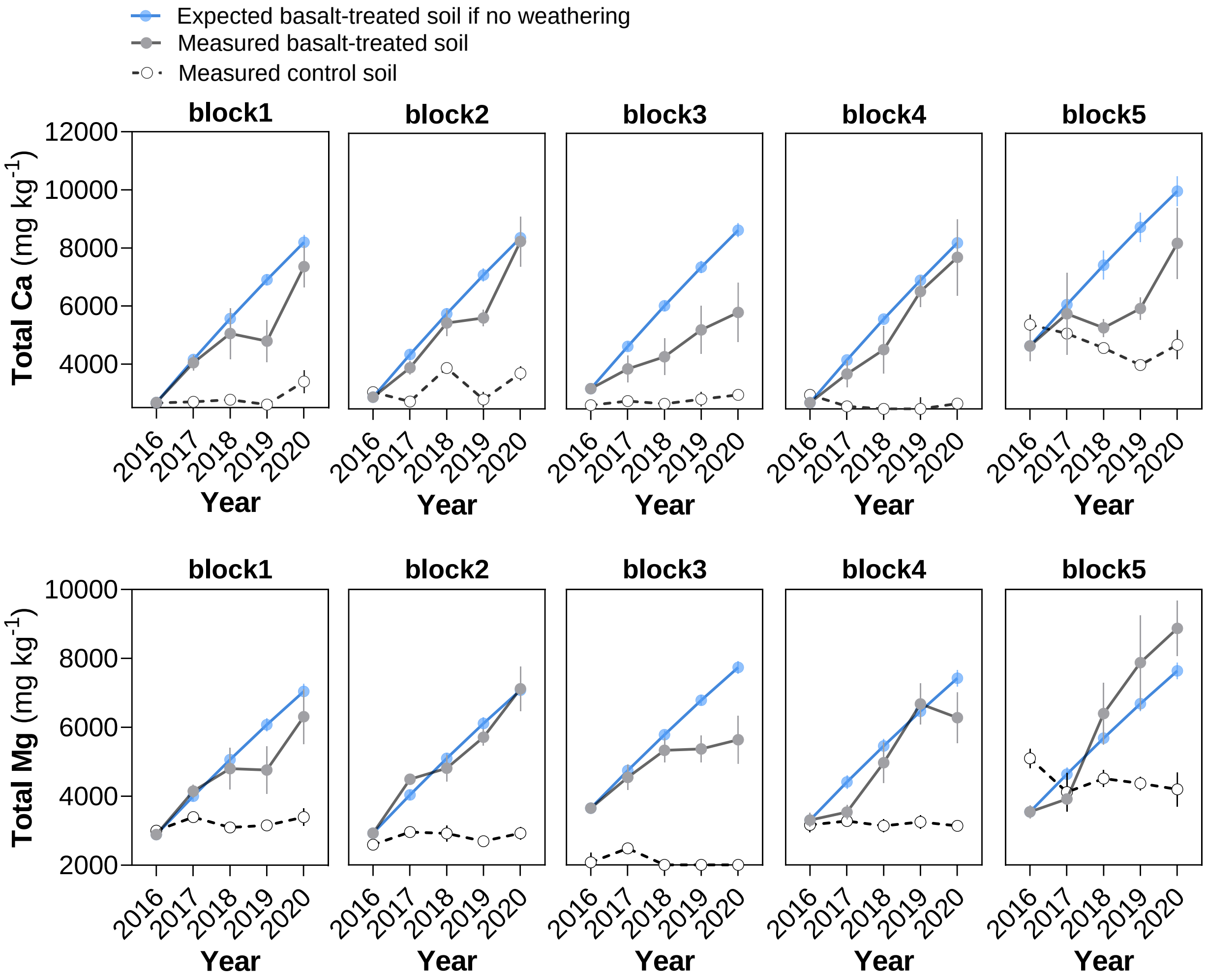

Fig. S3. Soil Ca and Mg pools across experimental blocks in response to EW. Measured total concentrations in soil are based on multi-acid digest of soils after each sample has been pre-extracted with 1M ammonium acetate and the exchangeable ion fraction has been removed. The line for the expected basalt-treated soil if there was no weathering is based on block and treatment specific soil end-member (based on pre-treatment 2016 measurements) and a basalt feedstock end-member. The mixing ratio was obtained by assuming mixing of 1000 t soil ha^{-1} (top 0-10cm) with 25 t basalt ha^{-1} yr^{-1} application rates. This figure is derived from the total basalt application of 50 t ha^{-1} yr^{-1}, assuming equal mixing by mechanized tilling in the top 20 cm, i.e., 25 t ha^{-1} yr^{-1} in the top 0-10 cm and the remaining 25 t ha^{-1} yr^{-1} in the bottom 10-20 cm.

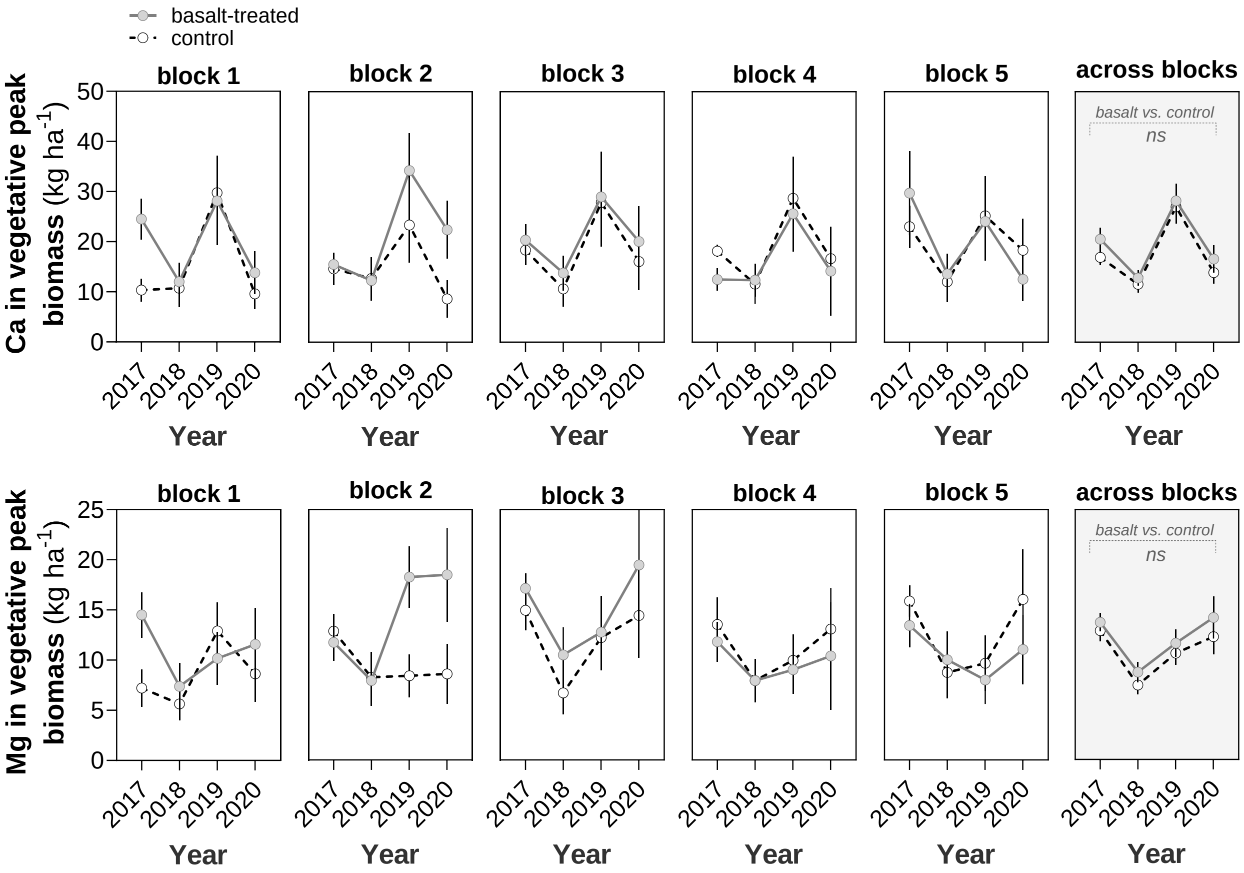

Fig. S4. Pools of Ca and Mg in peak vegetative biomass across experimental blocks in response to EW. Seed biomass was harvested in September of each trial year. The seed stocks were computed by multiplying metal concentration with seed biomass weight on a sample basis with each sample representing the pool of 0.36 m^2 quadrats with n = 2 per treatment per block for each trial year.

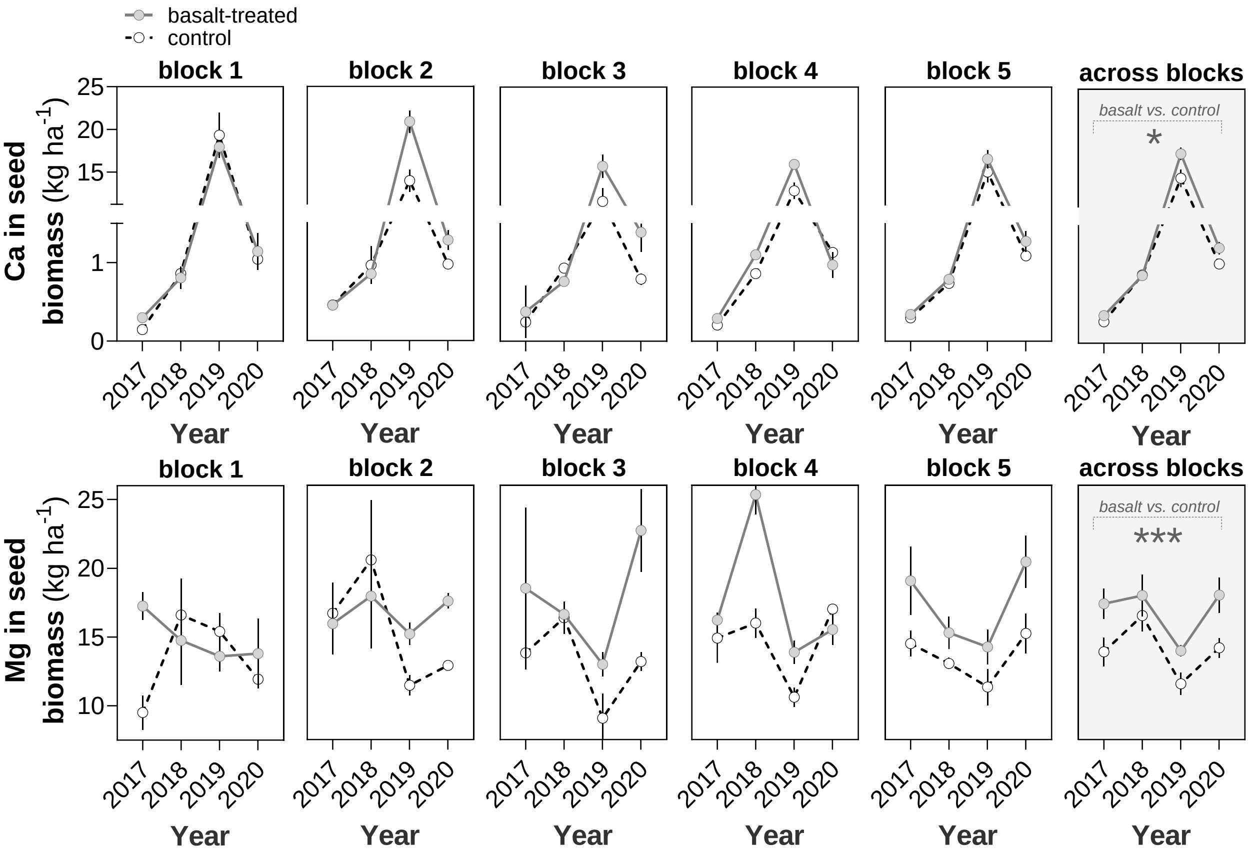

Fig. S5. Pools of Ca and Mg in seed biomass across experimental blocks in response to EW. Seed biomass was harvested in September of each trial year. The seed stocks were computed by multiplying metal concentration with seed biomass weight on a sample basis with each sample representing the pool of 0.36 m^2 quadrats with n = 2 per treatment per block for each trial year.

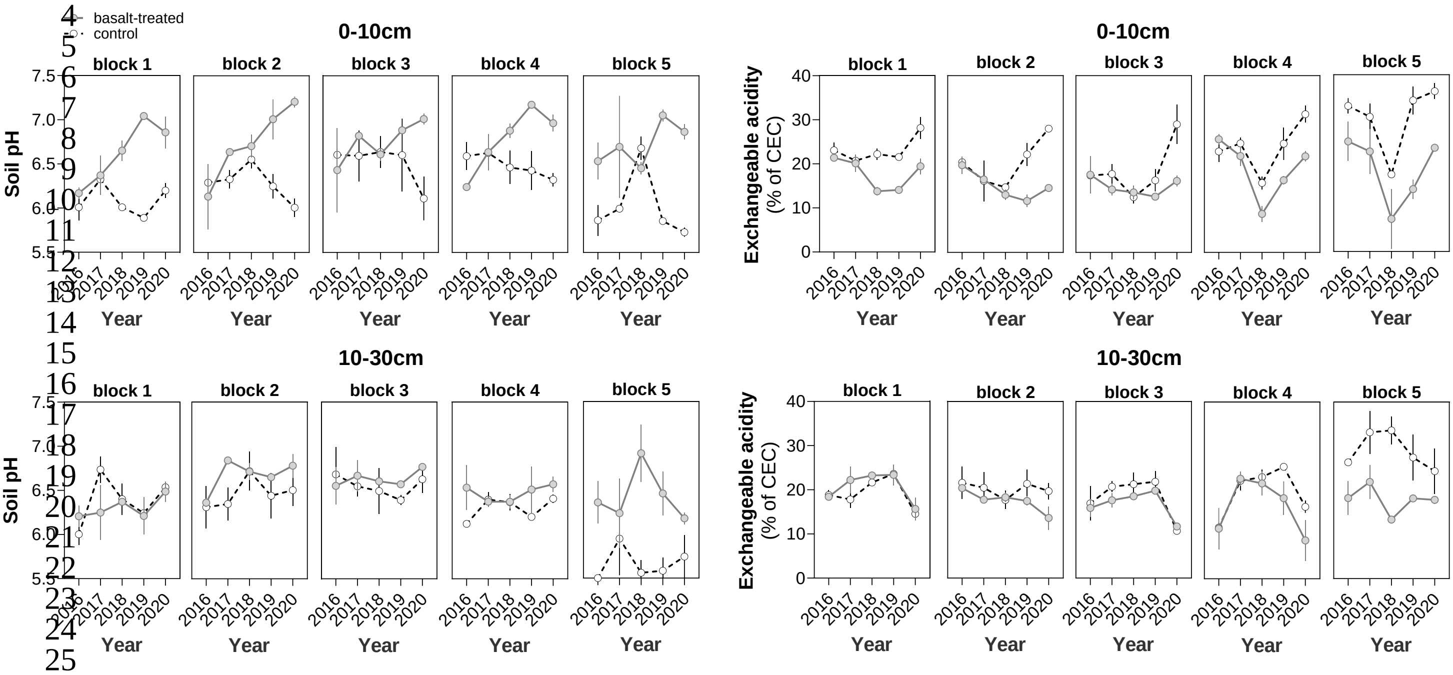

Fig. S6. Soil pH and percentage exchangeable acidity across experimental blocks in response to EW.

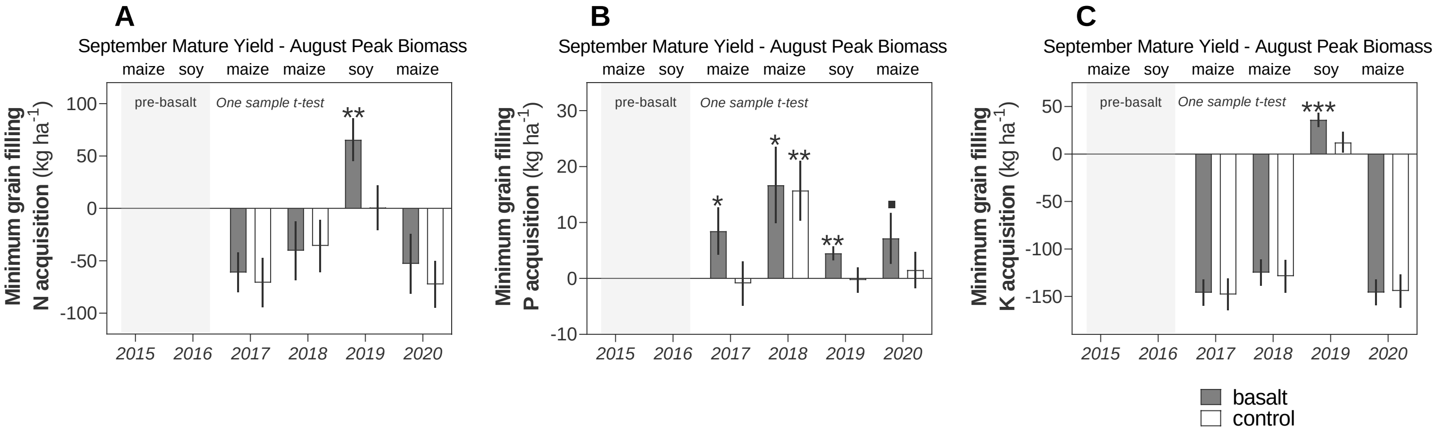

Fig. S7. Minimum acquisition of N, P, and K during the grain filling stage, mid-August to mid-September.

Acquisition of (**A**) nitrogen (N), (**B**) phosphorus (P) and (**C**) potassium (K) by crops during grain filling. Negative values indicate the biomass in mid-August supplied nutrients for grain biomass in mid-September via remobilization, or from soil and via N_2-fixation in the case of N for soybean. Positive values indicate that further nutrient acquisition was required to accommodate for grain biomass. The minimum acquisition scenario assumes 100% remobilization and transfer of N, P, and K from vegetative peak biomass to grain thus representing a conservative estimate of nutrient acquisition during the duration of the grain filling stage. Statistical tests: one tailed one sample t-test, ***$P < 0.001$, **$P < 0.01$, *$P < 0.05$, ▪$P < 0.10$.

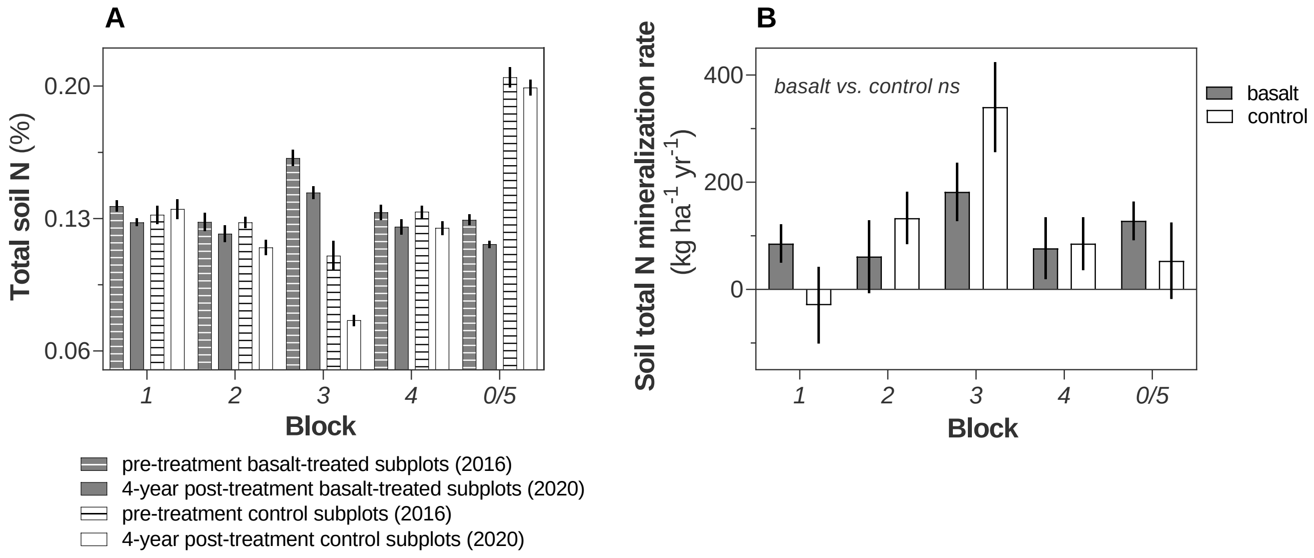

Fig. S8. Changes in soil total N stocks and mineralization rates between 2016 and 2020.

(A) total soil N and **(B)** mineralization rates calculated as difference between $2020_{block/treatment}$ – $2016_{block/treatment}$. Overall, mineralization rates are not significantly different between basalt-treated and control blocks (the mean for control plots and basalt-treated plots were used in the calculation of nitrogen use efficiency). The statistical test performed in B is a Two-way ANOVA with two-factors: block and treatment.

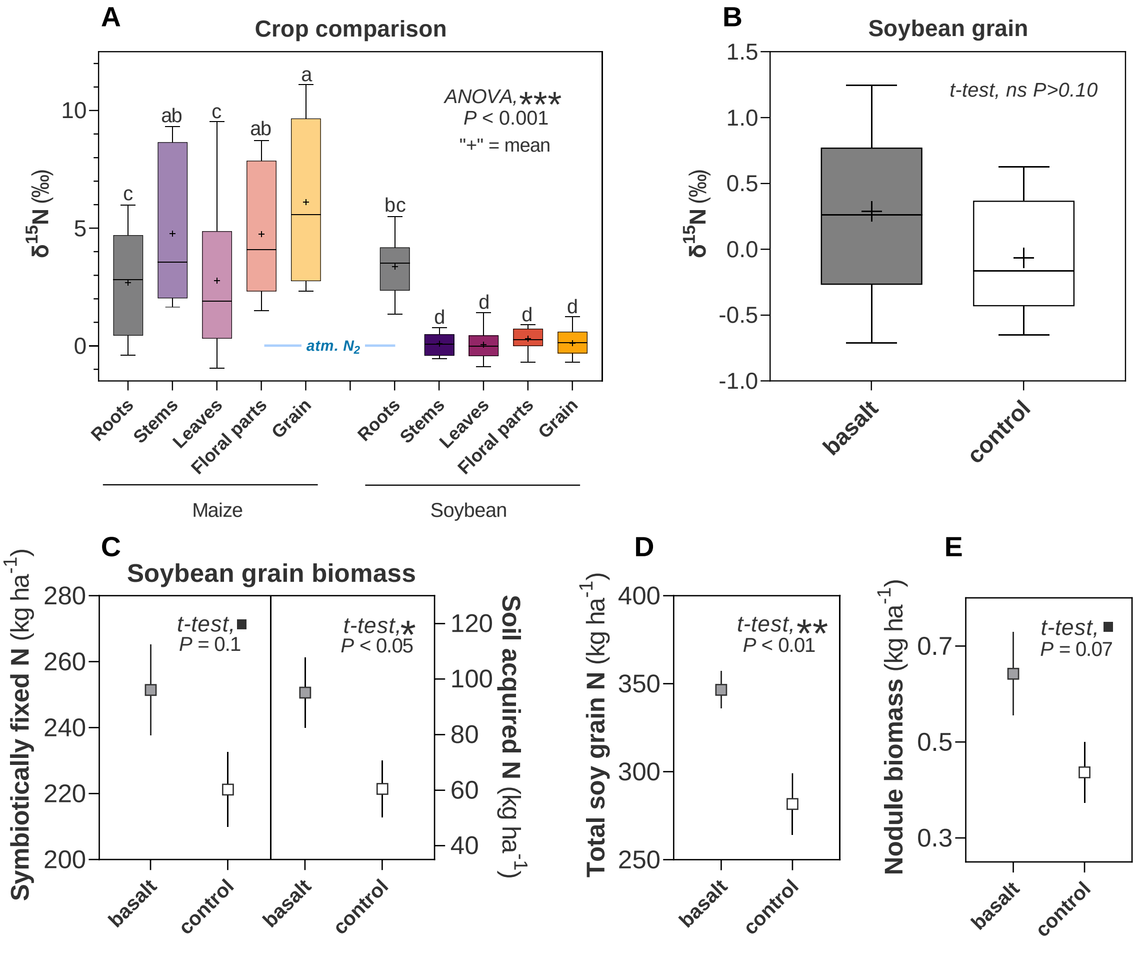

Fig. S9. δ^{15}N of plant tissues for differentiation of symbiotically fixed vs. soil acquired N in soybean grains in response to EW.

(**A**) Intercrop comparison shows that soybean organs (except roots) lower δ^{15}N values than maize, consistent with symbiotic N_2-fixation. **(B)** Comparison between δ^{15}N in basalt-treated soybean and control soybean plants. **C.** Trend towards higher symbiotically fixed N (~ +30 kg ha^{-1} in soybean biomass) and significantly higher soil acquired N (~ +35 kg ha^{-1} in soybean biomass). **(D)** Total grain N in basalt-treated vs. control soybean plants indicative of both higher N_2-fixation rates and NUE in EW-treated soybean and **(E)** nodule biomass response to EW. Statistical tests: ANOVA for A and t-tests for B-E. Error bars in C-E show SEM.

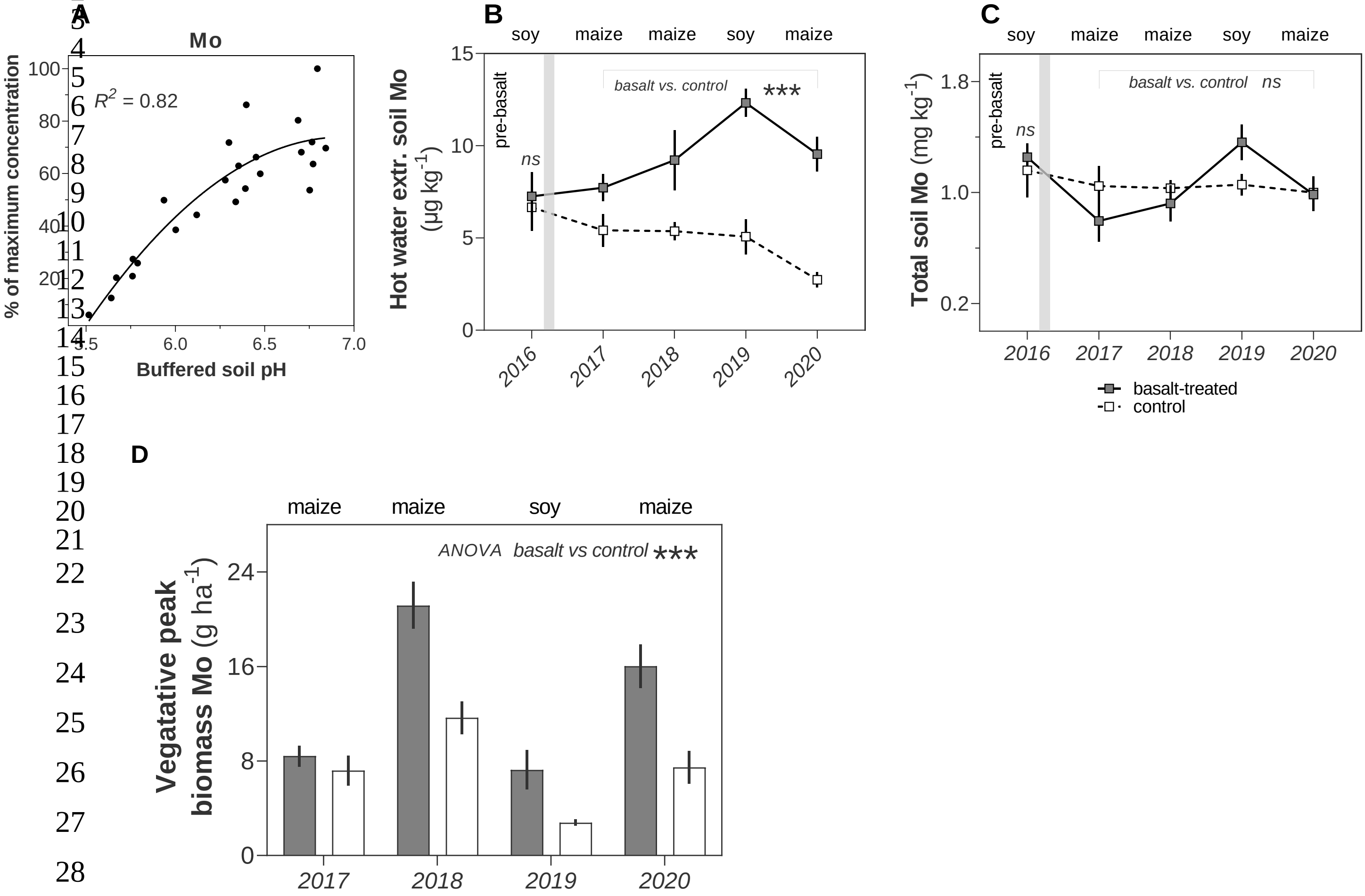

Fig. S10. Soil and vegetation molybdenum budgets (Mo) over time.

(**A**) Mo concentrations in soil solution are strongly dependent on soil pH as increasing pH promotes Mo desorption from soil particles, (**B)** plant available Mo, as assessed by hot water extractable Mo, increases with EW treatment over time as soil pH increases relative to controls and (**C)** Total Mo soil pool is not significantly different between EW-treated and control soils suggesting that the increased stocks of Mo in biomass do not deplete total soil stocks. Error bars = 1 s.e.m. Note change in units between B and C. (**D**) Vegetative biomass Mo stocks increase significantly with EW maize and soybean relative to controls.

Statistical tests: Two-way ANOVA, time point (year) as one factor, and basalt vs. control as the other main factor. Values shown are for the basalt vs. control main factor, *** $P < 0.001$.

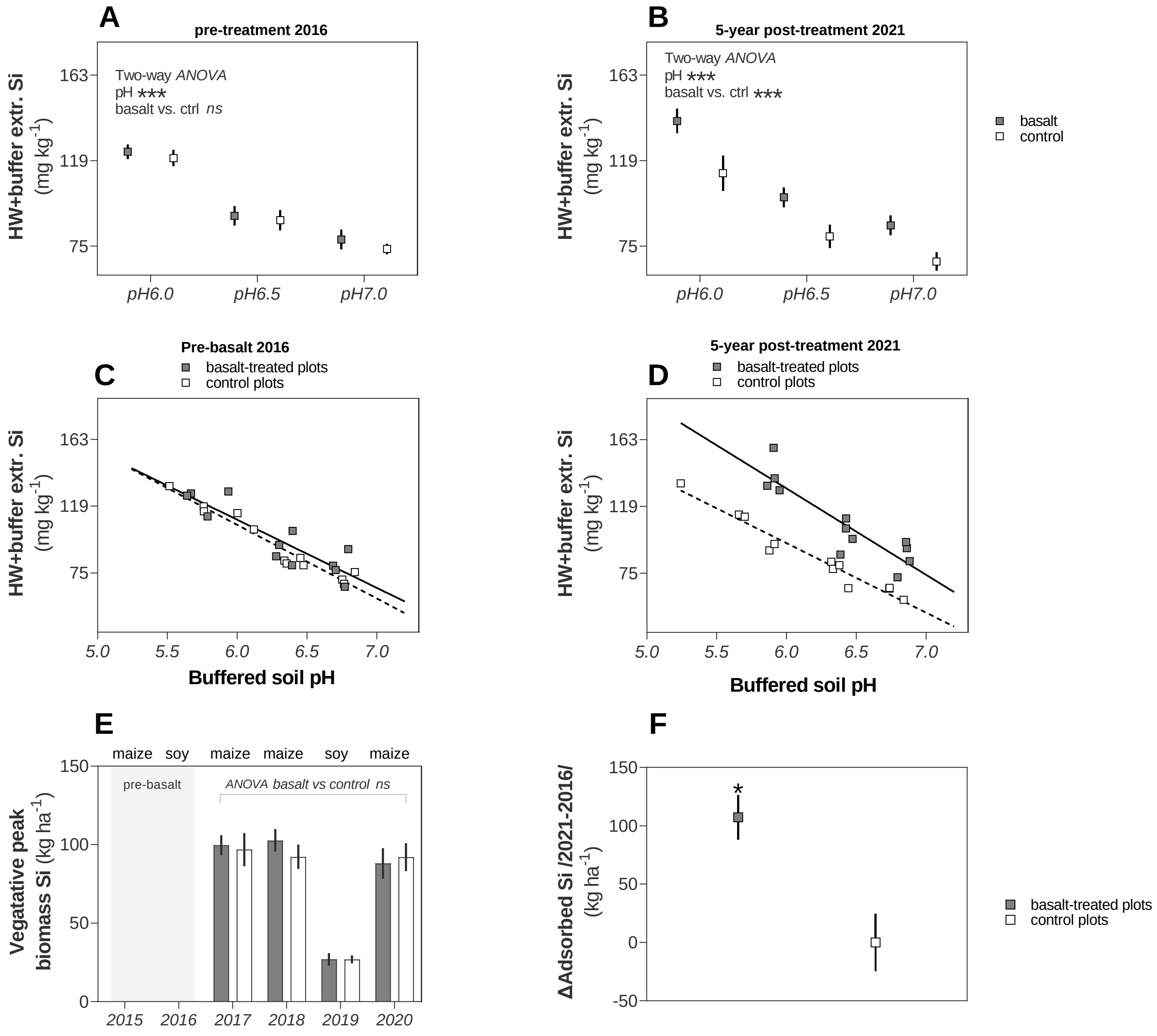

Fig. S11. Soil and plant silicon dynamics following EW.

(**A**) Pre-treatment (pre-basalt) comparison of adsorbed Si extracted at different pH buffered solutions. No difference between pre-treatment control and basalt-treated soils (2016), only a pH effect, with more silica is desorbed at low pH. (**B)** Five-year post-treatment comparison of adsorbed Si extracted at different pH buffered solutions with a significant increase in adsorbed silicon in basalt-treated soils compared to control soils. (**C)** Correlation between buffered soil pH and Si extracted in pre-treatment (2016) samples, (**D**) Correlation between buffered soil pH and Si extracted for basalt-treated and control soils. Basalt-treated soil contains more adsorbed silicon than control soils at any given soil pH value, (**E**) biomass pools of Si showing no effect of EW due to balancing of adsorption and release Si by basalt weathering. (**F**) Estimated Si adsorbed onto soil particles in basalt-treated versus control soils (two-tailed t-test, $P < 0.05$). These findings help explain the lack of significant difference in biomass Si stocks between basalt-treated and control crop biomass. Basalt weathering releases Si but the higher pH increases adsorption, with the two effects compensating each other.

.

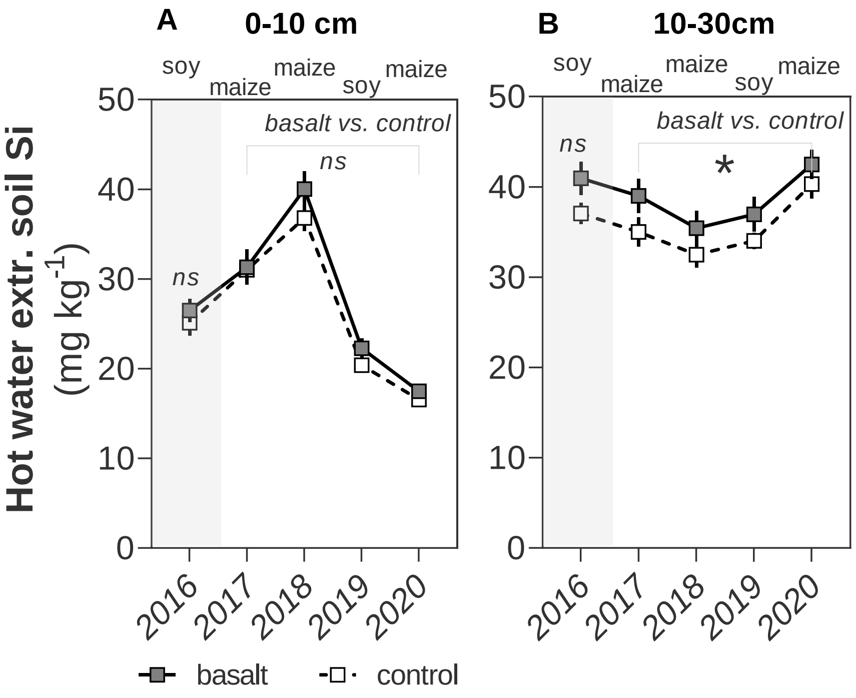

Fig. S12. Soil silicon (Si) in unbuffered hot water soil extracts at native soil pH.

(**A**) Soil silica (Si) at depth 0-10 cm and (**B**) and at 10-30 cm depth. Soil at the 0-10 cm depth is notably more depleted in Si than the 10-30 cm layer, consistent with uptake of crop Si at the 0-10 cm where root density is highest.

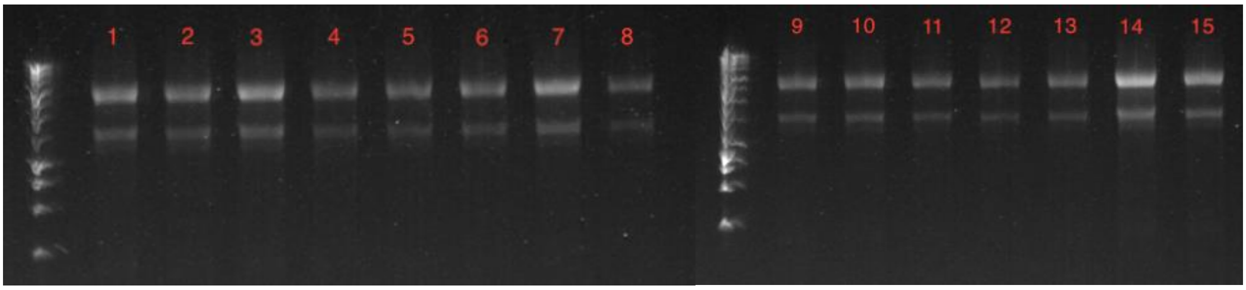

Fig. S13. Spot checks of the integrity of total RNA extracts from plant roots.

The presence of the characteristic 25S rRNA and 18S rRNA bands of plants in 15 randomly selected samples demonstrates that extracted RNA was of high integrity suitable for downstream applications. The 15 samples were checked on two separate 9-well gels using an RNA ladder.

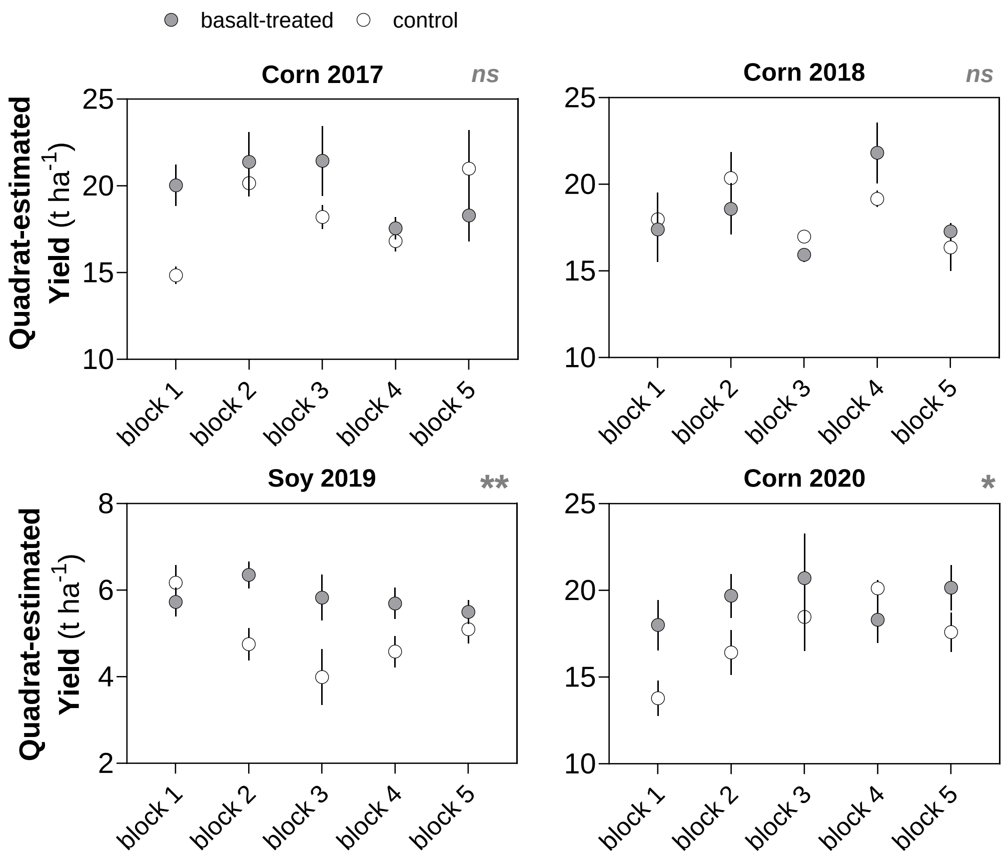

Fig. S14. Crop yield responses to EW by trial blocks and year. Years where the overall effect of basalt treatment was significant (unpaired two-tailed t-test, ** P < 0.01, * < 0.05, *ns* > 0.05) were 2019 (soybean) and 2020 (corn). The 0.75 × 0.75 m quadrats were 4 per treatment per block each corn year given n = 20 quadrats for both control and basalt-treated. In the soy year (2019), the replication quadrats were 2 per treatment per small block and 4 for block 5 to a total of n = 12 quadrats for both control and basalt-treated.

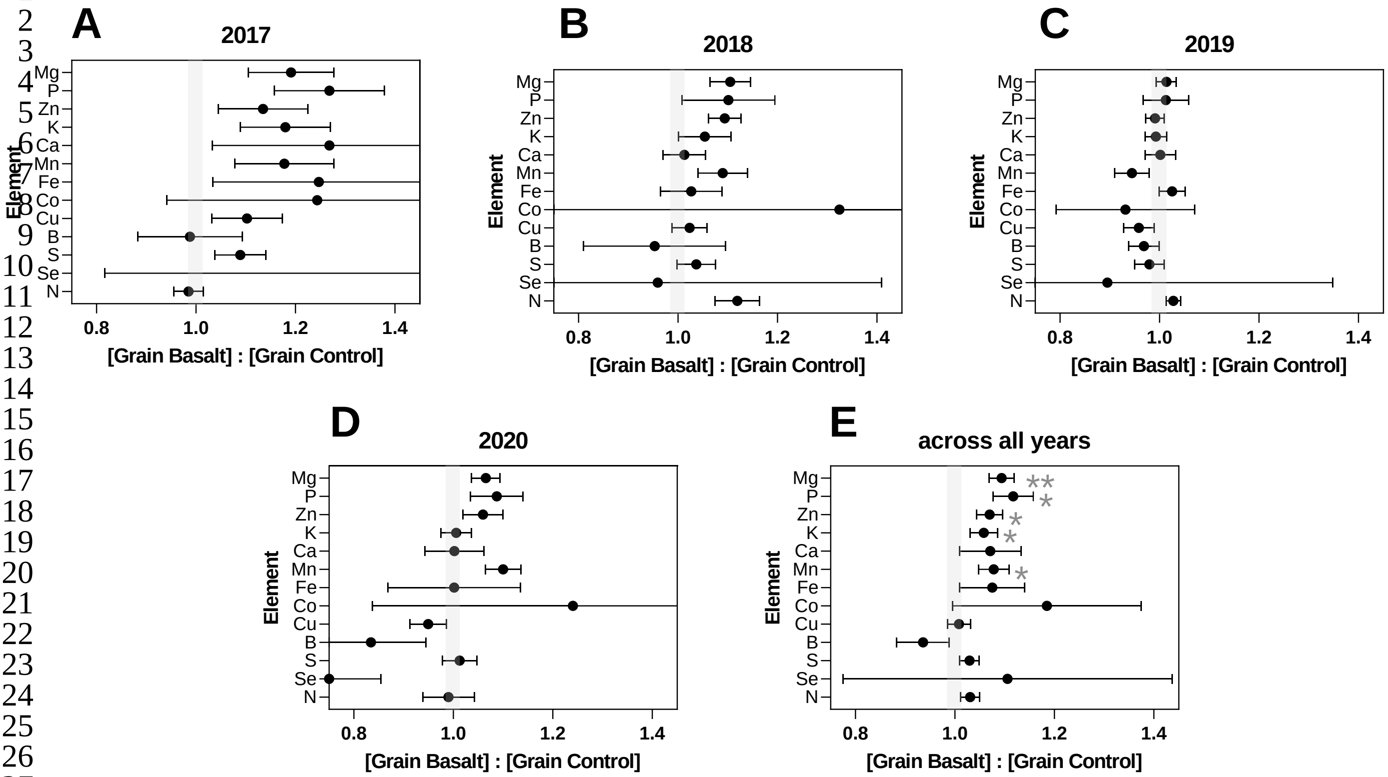

Fig. S15. Changes in grain concentration of macro- and micronutrients important for nutrition with EW.

Ratio between elemental concentrations in basalt-treated and control grains (**A**)-(**D**) 2017 – 2020 and (**E**) average across all years. E W significantly increases the grain concentrations of P, Mg, Zn, K, and Mn (One sample t-test, testing if ratio significantly greater than 1; $**P < 0.01$, $*P < 0.05$). The ratio of elemental concentration in basalt-treated and control grains were averaged for each of the four treatment years and mean and standard error of mean derived from the four annual ratio values. Standard deviation was calculated using error propagation rules. Note that except for marginal pattern in boron (B), EW increases yields without impoverishment of key nutrients.

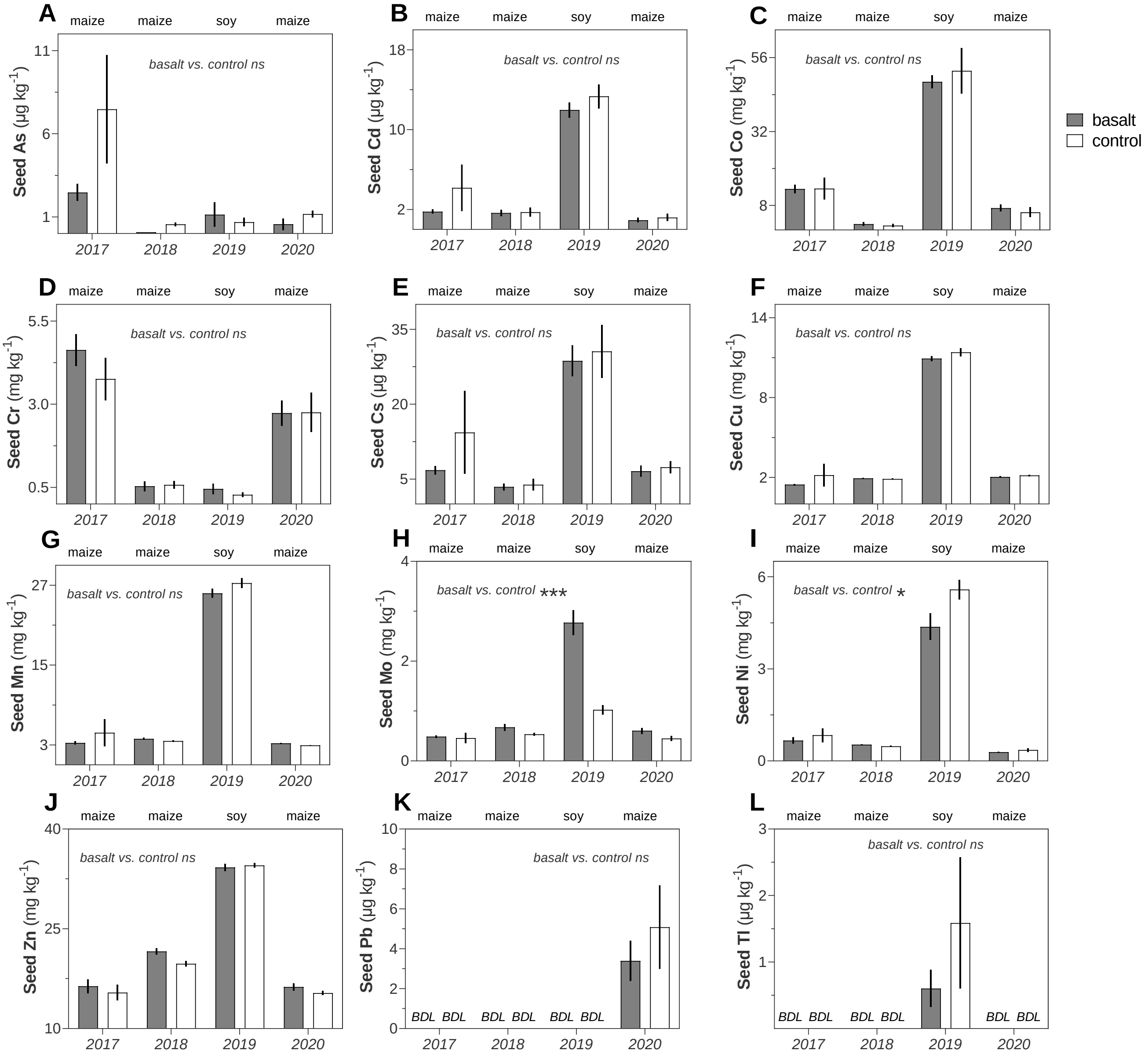

Fig. S16. Concentrations of trace metals in seeds from basalt-treated and control crops.

(A) Seed As concentration, (**B**) Cd concentration, (**C**) Co concentration (mg kg^{-1}); (**D**) Cr concentration (mg kg^{-1}), **(E)** Cs concentration, (**F**) Cu concentration (mg kg^{-1}), (**G**) Mn concentration (mg kg^{-1}), (**H**) Mo concentration (mg kg^{-1}) (**I**) Ni concentration (mg kg^{-1}), (**J**) Zn concentration (mg kg^{-1}), (**K**) Pb concentration, (**L**) Tl concentration. A two-way ANOVA test was carried out with two-factors, year (2017, 2018, 2019, and 2020) and treatment (basalt vs. control). Legend to statistical tests for main factor treatment: Two-way ANOVA, *** $P < 0.001$, ** $P < 0.01$, * $P < 0.05$, ^{ns} $P > 0.05$. Bars indicate SEM. Replication $n = 10$ samples per treatment per year, except in cases where values were below detection limits (BDL). BDL is placed for samples with concentrations below detection limits. In the case of Pb and Tl only one sampling season had enough replicates ($n \geq 3$ per treatment). Consequently, for these elements a two-tailed t-tests was performed instead of an ANOVA test. All values are based on seed dry biomass. Full names of elements: As = arsenic, Cd = cadmium, Cr = chromium, Cs = caesium, Cu = copper, Mn = manganese, Mo = molybdenum, Ni = nickel, Zn = zinc, Pb = lead, Tl = thallium.

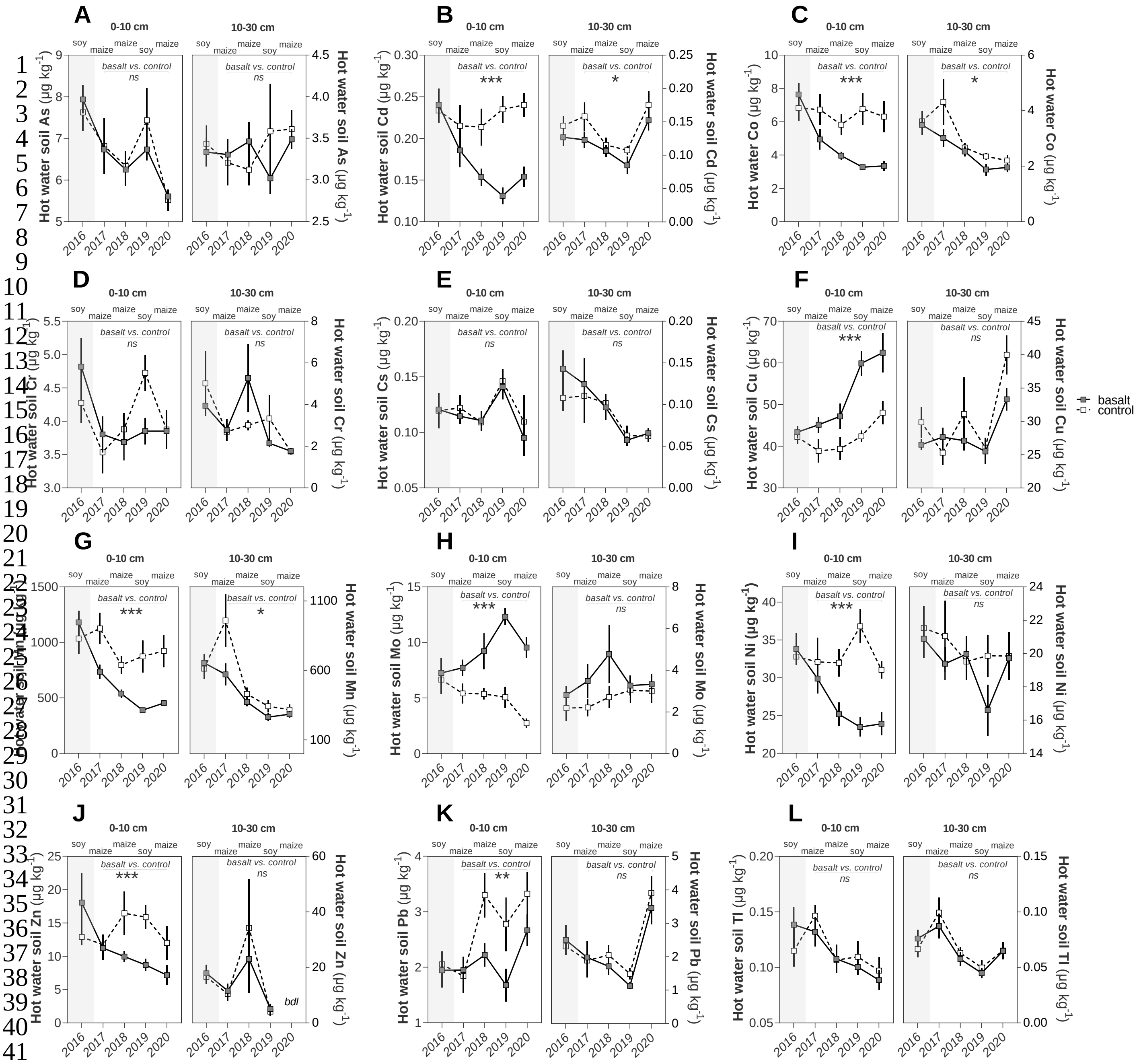

Fig. S17. Dynamics of trace metals in the soil pore water across depths.

(**A**) arsenic, (**B)** cadmium, (**C)** cobalt, (**D)** chromium, (**E)** caesium, (**F**), copper, (**G**) manganese, **(H)** Molybdenum, (**I**) nickel, (**J**) zinc, (**K**) lead and (**L)** thallium. A two-way ANOVA test was carried out with two-factors, year (2017, 2018, 2019, and 2020) and treatment (basalt vs. control). Legend to statistical tests for main factor treatment: Two-way ANOVA, *** $P < 0.001$, ** $P < 0.01$, * $P < 0.05$, ^{ns} $P > 0.05$. $n = 10$ replicates for each treatment (basalt and control) for each depth (0-10 and 10-30cm) and for each year (2016-2020). Error bars represent s.e.m.

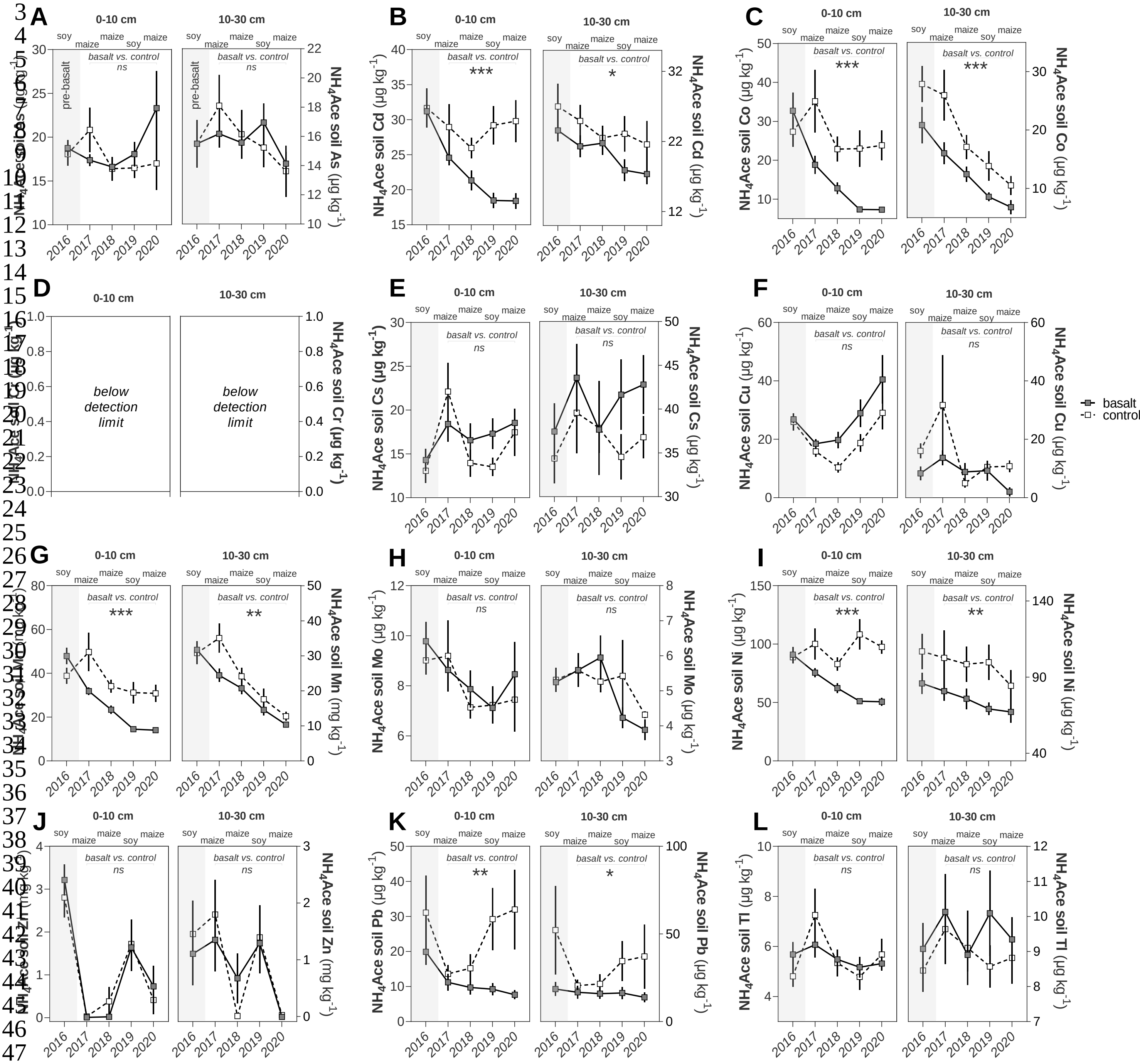

Fig. S18. Dynamics of trace metals in the soil exchangeable pool across depths. (**A**) arsenic, (**B**) cadmium, (**C**) cobalt, (**D**) chromium, (**E)** caesium, (**F**) copper, (**G**) manganese; (**H**) molybdenum, (**I**) nickel, (**J**) zinc, (**K**) lead and (**L** thallium. A two-way ANOVA test was carried out with two-factors, year (2017, 2018, 2019, and 2020) and treatment (basalt vs. control). Legend to statistical tests for main factor treatment: Two-way ANOVA, *** $P < 0.001$, ** $P < 0.01$, * $P < 0.05$, ^{ns} $P > 0.05$. NH_4Ace stands for 1M ammonium acetate solution (pH 7.0) that was used to extract the soil exchangeable nutrient fraction. $n = 10$ replicates for each treatment (basalt and control) for each depth (0-10 and 10-30cm) and for each year (2016-2020). Error bars represent s.e.m.
